# The antimalarial drug amodiaquine stabilizes p53 through ribosome biogenesis stress, independently of its autophagy-inhibitory activity

**DOI:** 10.1038/s41418-019-0387-5

**Published:** 2019-07-08

**Authors:** Jaime A. Espinoza, Asimina Zisi, Dimitris C. Kanellis, Jordi Carreras-Puigvert, Martin Henriksson, Daniela Hühn, Kenji Watanabe, Thomas Helleday, Mikael S. Lindström, Jiri Bartek

**Affiliations:** 1 Science for Life Laboratory, Division of Genome Biology, Department of Medical Biochemistry and Biophysics, Karolinska Institutet, S-171 21 Stockholm, Sweden; 2 Science for Life Laboratory, Department of Oncology-Pathology, Karolinska Institutet, S-171 76 Stockholm, Sweden; 3 Danish Cancer Society Research Center, DK-2100 Copenhagen, Denmark; 4 Weston Park Cancer Centre, Department of Oncology and Metabolism, University of Sheffield, Sheffield, S10 2RX UK

**Keywords:** Cell biology, Cancer

## Abstract

Pharmacological inhibition of ribosome biogenesis is a promising avenue for cancer therapy. Herein, we report a novel activity of the FDA-approved antimalarial drug amodiaquine which inhibits rRNA transcription, a rate-limiting step for ribosome biogenesis, in a dose-dependent manner. Amodiaquine triggers degradation of the catalytic subunit of RNA polymerase I (Pol I), with ensuing RPL5/RPL11-dependent stabilization of p53. Pol I shutdown occurs in the absence of DNA damage and without the subsequent ATM-dependent inhibition of rRNA transcription. RNAseq analysis revealed mechanistic similarities of amodiaquine with BMH-21, the first-in-class Pol I inhibitor, and with chloroquine, the antimalarial analog of amodiaquine, with well-established autophagy-inhibitory activity. Interestingly, autophagy inhibition caused by amodiaquine is not involved in the inhibition of rRNA transcription, suggesting two independent anticancer mechanisms. In vitro, amodiaquine is more efficient than chloroquine in restraining the proliferation of human cell lines derived from colorectal carcinomas, a cancer type with predicted susceptibility to ribosome biogenesis stress. Taken together, our data reveal an unsuspected activity of a drug approved and used in the clinics for over 30 years, and provide rationale for repurposing amodiaquine in cancer therapy.

## Introduction

Ribosome biogenesis has emerged in recent years as a potent therapeutic target against cancer. Fast-dividing malignant cells require enhanced ribosome production to meet the increased needs for protein synthesis to sustain the elevated metabolism and proliferation [1]. High rate of ribosome biogenesis is strongly associated with poor prognosis across many cancer types [2, 3], and it is commonly regulated by oncogenic signaling pathways including c-MYC [4], RAS-RAF-ERK [5], and PI3K–mTOR [6].

Transcription of ribosomal RNA (rRNA) is carried out exclusively by the RNA polymerase I (Pol I) inside the nucleolus. This process requires the presence of the upstream binding factor UBF on the active ribosomal DNA (rDNA) genes, enabling the recruitment of the pre-initiation complex (PIC) and Pol I components to the rDNA gene promoter. Each rDNA gene codes for the 47S rRNA precursor, which is further processed to form the 18S, 5.8S and 28S mature rRNAs. In parallel, RNA polymerase III transcribes the 5S rRNA in the nucleoplasm, while RNA polymerase II transcribes the mRNAs of the ribosomal proteins (RPs). Mature 5S rRNA and RPs are imported to the nucleolus and preassembled together with the 18S, 5.8S, and 28S RNAs to form the 60S and 40S ribosomal subunits. Subsequently, mature ribosomes are formed in the cytoplasm through a series of export and maturation processes [7].

Beside the deleterious effects of actinomycin D on rRNA synthesis [8], new ribosome biogenesis inhibitors have emerged. CX-5461 was described originally as an inhibitor of Pol I leading to activation of p53 due to ribosome biogenesis stress [4], however, new evidence has shown that it also stabilizes DNA G-quadruplexes, induces DNA damage, interferes with DNA replication [9] and activates ATM/ATR signaling [10]. Strikingly, the recently introduced compound BMH-21 stands out as a drug that triggers proteasomal degradation of the catalytic subunit of Pol I, RPA194 (POLR1A), and thereby activates p53 in the absence of DNA damage [11, 12]. Interestingly, the antineoplastic drug oxaliplatin, included in the clinical FOLFOX regimen and first-line treatment of colorectal cancer (CRC), exerts its chemotherapeutic effect predominantly through ribosome biogenesis stress rather than through DNA crosslinking, as its analog cisplatin [13]. These findings strongly support the rationale for developing new ribosome biogenesis inhibitors against CRC.

The repurposing of FDA-approved compounds is increasingly becoming an attractive strategy to identify new oncological applications. This approach harnesses advantages of “old” drugs: clinical safety, shorter times for drug development, and reduced investment at early stages [14]. Recently, we have provided an example of drug repurposing in oncology by providing epidemiological support, identifying the molecular target, mechanism of action and the active anticancer metabolite of the old and safe alcohol-abuse drug disulfiram [15].

The antimalarial drug amodiaquine (AQ) induces similar gene expression perturbation to that induced by the BMH family of ribosome biogenesis inhibitors, suggesting similar mechanisms of action [16]. AQ and its analog chloroquine (CQ) are members of the 4-aminoquinolines family of antimalarial drugs. Although AQ and CQ are similarly potent as autophagy inhibitors [17, 18], only AQ triggers p53 stabilization [18, 19] and displays higher cytotoxicity than CQ at equimolar concentrations [18]. However, the mechanisms underlying these differences remain unknown. Here we report the characterization of the FDA-approved drug AQ as a ribosome biogenesis inhibitor and promising anticancer compound.

## Material and methods

### Cell culture

Cell lines and culture conditions are described in Supplementary Table S1. Briefly, cells were reseeded 2–3 times per week, as soon as the monolayer became preconfluent or early confluent. Tests for mycoplasma detection were performed monthly. To generate the RKO ATM-knockout cells the following gRNA sequences (2.1) 5′-CACCGTGATAGAGCTACAGAACGAA-3′ and (6.3) 5′-CACCGAAACAATTAAACATCTAGAT-3′ were cloned into PX458 vector (Addgene #48138) and verified by sequencing. Three days after plasmid transfection, GFP-positive cells were sorted and knockout/absence of ATM was confirmed by immunoblots analysis of single clones.

### Immunoblotting

Subconfluent cells were directly lysed in RIPA buffer (Thermo, PI-89901) plus protease (cOmplete ULTRA, code 05892970001, Roche) and phosphatase inhibitors (PhosSTOP, code 04906837001 Roche) and sonicated during 3–5 cycles of 30 s on and 30 s off, in a Bioruptor^®^ (Diogenode). Protein quantification was performed with the DC™ Protein Assay Kit II (Bio-Rad, 5000112). Five to 20 μg was boiled in Laemmli sample buffer during 5 min at 95 °C, loaded onto SDS-PAGE gels and transferred to nitrocellulose or PVDF membranes. Chemiluminiscence signal was detected using SuperSignal™ West Dura (Thermo, 34076). Images were acquired with an Amersham Imager 600 scanner. Antibodies and their applications are listed in Supplementary Table S2.

Quantitative immunoblot was performed with Wes™ following manufacturer instructions (Protein Simple). Briefly, cells were lysed with RIPA, sonicated and analyzed. RPA194 levels were quantitated and normalized using β-actin as internal control.

### Chemicals

Chemicals used in this study are listed in the Supplementary Table S3. Compounds were dissolved in dimethyl sulfoxide (DMSO) or water according to the vendor’s instructions. Stock dilutions were aliquoted and stored at −20 °C. Resazurin stock solution was prepared by dissolving resazurin sodium salt powder in sterile DPBS, in a final concentration of 1 mg/mL. Resazurin working solution was prepared fresh right before use by diluting the stock solution in cell medium, to a final concentration of 20 μg/mL. Silver nitrate solution was also prepared fresh before use by dissolving the powder in miliQ water to a final dilution of 0.5 g/mL.

### Immunofluorescence and imaging

Cells grown in 96-wells imaging plates were fixed in 4% formaldehyde for 15 min at room temperature, washed with PBS, permeabilized in PBS 0.5% Triton X-100 during 10 min and blocked with PBS 3% bovine serum albumin during 30 min. Cells were sequentially incubated with the primary (over night) and secondary antibody (120 min), stained with Hoechst 2 μM during 15 min. Images were acquired using an IN Cell Analyzer 2200 (GE Healthcare). Image segmentation and foci/vesicles quantification was performed using Cell Profiler software [20].

### AgNOR staining

Cells were seeded in 8-well Chambered Cell Culture Slides (Falcon) at a density of 10^4^ cells/well. A vehicle well was included, containing cells treated with DMSO if required. The cells were fixed with 2% glutaraldehyde in PBS for 10 min at room temperature, following two sequential washing steps with PBS and dH_2_O, respectively. Next, cells were incubated with a second fixing solution (methanol:acetic acid 3:1) for 5 min and washed thoroughly with dH_2_O. Further, 200 μL of silver nitrate colloidal solution (a 0.5 g/mL silver nitrate aqueous solution was diluted in 2% gelatin and 1% formic acid, in a proportion of 2:1) was added to each well and incubated in the dark for 20 min, at room temperature. Then, the slides were washed vigorously with dH_2_O and coverslips were mounted using Prolong Gold (Invitrogen, P36930). AgNOR stained foci appeared as black brown dots within the nucleus were evaluated under a light microscope at (×20 and ×40) magnification (Olympus BX53). The photos were taken with a digital color camera (Olympus DP73).

### Fluorescent intercalator displacement (FID) assay

The FID assay was conducted as previously described elsewhere [21]. Deoxyoligonucleotide oligo hairpins containing an 8-bp stem region with random DNA sequences with increasing GC content (Supplementary table S6) were purchased from Sigma-Aldrich. Ethidium bromide (EtBr; 6 μM) was added to each well in sodium acetate buffer (35 mM NaOAc, 162.8 mM NaCl, 1.75 mM EDTA, pH 5.0), followed by hairpin DNA (1.5 μM), and 2 μM of each compound tested, diluted in sodium acetate buffer containing 2% DMSO. For each experiment, the ratio of EtBr to hairpin DNA was adjusted to 1 M equivalent EtBr per 2 DNA base pairs. The 0% control wells contained neither hairpin DNA nor test compounds, while the 100% control wells contained hairpin DNA and EtBr but no test compounds. All assays were conducted in triplicate.

### siRNA knockdown

Cells were transfected with 20 nM siRNA using Lipofectamine™ RNAiMAX during 6 h in Opti-MEM (Gibco, 31985070) and 18 additional hours in regular DMEM. For RPL5, siRNA 5′-AAGGUUGGCCUGACAAAUUAUUU-3′ sequence was used. ON-TARGETplus non-targeting pool (D-001810-10-20) and SMARTpool: ON-TARGETplus RPL11 siRNA (L-013703-00) were purchased from Dharmacon. Flexitube siRNAs Hs_TP53_8 and Hs_TP53_9 were purchased from Qiagen.

### Viability assay and GI50

Cell lines were seeded at 500 cells/well or 1000 cells/well (for BJ cells) in 384-well plates (#3764, Corning^®^, Sigma Aldrich) 24 h prior to treatment, in a total volume of 35 μL, by using a MultiFlo FX Multi-Mode Dispenser (BioTek, AH Diagnostics Sweden, SE-169 70). Plates were incubated at room temperature for 20 min to minimize the edge effect before placing them in a 37 °C incubator. Master plates (384 V-bottom polypropylene plates, #781280, Greiner) containing the single compounds and/or their combinations were manually prepared at an 8× dilution in media, according to the experimental layout. For the dose response curves experiments twofold serially diluted concentrations of the single compounds were used. Five microliters of compound was transferred from the master plate to the cells to a final volume of 40 μL using VIAFLO 384 (Integra Biosciences, INTEGRA Biosciences AG, CH-7205, Switzerland). The inhibitory effect of the single agents and/or their combinations was determined by the resazurin assay. After 96 h incubation with compounds, the compound-containing medium was aspirated using a HydroSpeed microplate washer (Tecan) leaving a residual volume of 20 µL/well, and 20 µL of resazurin working solution was added using MultiFlo FX, to a final concentration of 10 µg/mL and a final volume of 40 µL. The plates were further incubated for 2 h and the emitted fluorescence was measured with a microplate reader using the 560 nm excitation/590 nm emission filter set (Tecan Infinite M1000 Pro, Männedorf, Switzerland). The assay was performed with three technical and three biological recplicates including vehicle controls (DMSO-treated cells), negative no-cell controls, and background subtraction controls (media and compound without cells) to ensure that the tested compounds are not autofluorescent at the applied wavelength. Results were statistically analyzed and normalized as % viability compared with the vehicle control, after background subtraction using GraphPad Prism 7.0 (Graph Pad Software Inc., San Diego, CA). Single-agent dose-effect curves were plotted, by applying three-parametric nonlinear regression. GI50 values were automatically calculated by the software.

### Transmission electron microscopy (TEM)

TEM was performed in EMil core facility at Karolinska Institutet. Cells were fixed in 2.5% glutaraldehyde in 0.1 M phosphate buffer, pH 7.4 at room temperature for 30 min. The cells were scraped and transferred to Eppendorf tube and further fixed over night in the refrigerator. After fixation cells were rinsed in 0.1 M phosphate buffer and centrifuged. The pellets were then postfixed in 2% osmium tetroxide (TAAB, Berks, England) in 0.1 M phosphate buffer, pH 7.4 at 4 °C for 2 h, dehydrated in ethanol followed by acetone and embedded in LX-112 (Ladd, Burlington, Vermont, USA). Ultrathin sections (~50–60 nm) were cut by a Leica EM UC 6 (Leica, Wien, Austria). Sections were contrasted with uranyl acetate followed by lead citrate and examined in a Hitachi HT 7700 (Tokyo, Japan) at 80 kV. Digital images were taken by using a Veleta camera (Olympus Soft Imaging Solutions, GmbH, Münster, Germany). Cells were trypsinized, harvested, and pelleted before fixation with glutaraldehyde.

### Quantitative real-time reverse transcription PCR

Total RNA was extracted with the PureLink™ RNA Mini Kit (ThermoFisher) following manufacturer’s instructions, and qPCR was performed using the Power SYBR^®^ Green RNA-to-CT™ 1-Step Kit (4389986, ThermoFisher) in a QuantStudio 5 PCR System. Cycling parameters: Reverse transcription at 48 °C for 30 min, initial denaturation at 95 °C for 30 s, and 40 cycles of 95 °C for 15 s and 62 °C for 60 s. Melt curve analysis: 95 °C for 15 and a gradual increase in temperature to 95 °C (0.075 °C/s). Triplicate treatment samples and three technical replicates per sample were analyzed. Relative quantity was analyzed with the ΔΔCt method using ACTB mRNA as internal normalizer. Primer sequences are listed in Supplementary Table S4. Primers sequences for 47S rRNA processing were obtained from [22].

### Chromatin immunoprecipitation

U2OS cells were fixed with 1% formaldehyde and processed using the Pierce Agarose ChIP Kit (26156, Thermo Scientific) following manufacturer’s instructions. qPCR was performed using Power SYBR™ Green PCR Master Mix (ThermoFisher). Primer sequences are listed in Supplementary Table S5 and were obtained from [12]. Analysis of the qPCR data was performed using the fold enrichment method, adjusting nonspecific amplification with the Mock IgG.

### RNA sequencing and gene ontology analysis

RNA sequencing was performed by National Genomics Infrastructure at Science for Life Laboratory Stockholm, Sweden. RNA quality was assessed by electrophoresis using a TapeStation Instrument (Agilent). Only samples with a RIN above nine were included. Library was prepared with Illumina TruSeq Stranded mRNA using Poly-A selection. Samples were sequenced on HiSeq2500. RNAseq data are available in the ArrayExpress database (http://www.ebi.ac.uk/arrayexpress) under accession number E-MTAB-7616. File processing was carried by NGI-RNAseq bioinformatics pipeline. Briefly, Fastq files where aligned using STAR (v2.5.1b) in two pass mode against reference genome (Homo sapiens, GRCh37). Read counting was performed using StringTie (v1.3.3) to further use prepDE python script. Differential expression analysis was performed using DESeq2 (v1.16.1). For further comparisons across the different treatments, gene list where filtered using a 0.5 linear fold change. Threshold and 0.001 adjusted *p*-value were performed on RStudio (v0.99.489, R 3.4.1).

ClueGO is a cytoscape plug in app that visualizes nonredundant biological terms for large clusters of gene sets in a functionally grouped network [23]. In our study, the enrichment analysis of gene-GO terms and pathways was perfomed using GO term Biological Process-EBI-QuickGO-GOA, KEGG pathways, REACTOME pathways and WikiPathways, date version 20-11-2017. Clusters with a *p*-value < 0.001 were included in figure, with the exception of BMH-21 specific genes, where was applied a Bonferroni correction and *p*-value < 0.05.

### Chemical synthesis

Starting materials and intermediates used in the synthesis of compounds described herein are commercially available from Sigma-Aldrich, Enamine, and Combi-Blocks. Flash column chromatography was performed in a ISCO combi flash system using Merck silica gel 60 Å (40−63 mm mesh). 1H NMR spectra were recorded on a Bruker DRX-400. Chemical shifts are expressed in parts per million (ppm) and referenced to the residual solvent peak. Analytical HPLC-MS was performed on an Agilent MSD mass spectrometer connected to an Agilent 1100 system with: Method acidic pH, Column ACE 3 C8 (50 mm × 3.0 mm), H_2_O (+0.1%TFA), and MeCN were used as mobile phases at a flow rate of 1 mL/min, with a gradient time of 3.0 min. Detection was made by UV using the 180−305 nM range and MS (ESI+). *Synthesis of 7-chloro-N-{3-[(diethylamino)methyl]phenyl}quinolin-4-amine (Amodiaquine analog*; *deshydroxy-amodiaquine; DH-AQ):* A mixture of 3-[(diethylamino)methyl]aniline (64 mg, 0.36 mmol), 4,7-dichloroquinoline (71 mg, 0.36 mmol), ethanol (2 mL), and 12 M HCl (30 µL, 0.36 mmol) was heated in the micro wave oven at 150 °C for 30 min. The solvent was removed in a rotavapor. Water (3 mL) and 5 M NaOH (0.5 mL) were added to the crude material and the mixture was extracted with dichloromethane (2 mL). The organic phase was dried over Na_2_SO_4_ and the product was purified by column chromatography on silica (10 g, 0–100% ethylacetate in iso-hexane as eluent) to generate 7-chloro-N-{3-[(diethylamino)methyl]phenyl}quinolin-4-amine (25 mg, 20%) [M + H] + *m/z* 340; 1H NMR (400 MHz, DMSO-d6) ppm 0.98 (t, *J* = 7.1 Hz, 6H), 2.44–2.50 (m, 4H, partly under solvent peak), 3.54 (s, 2H), 6.91 (d, *J* = 5.4 Hz, 1H), 7.07–7.11 (m, 1H), 7.20–7.26 (m, 1H), 7.30–7.39 (m, 2H), 7.56 (dd, *J* = 9.0, 2.4 Hz, 1H), 7.89 (d, *J* = 2.2 Hz, 1H), 8.40–8.47 (m, 2H), 9.10 (s, 1H). *Synthesis of 7-chloro-N-[3-(pyrrolidin-1-ylmethyl)phenyl]quinolin-4-amine (Amopyroquine analog: deshydroxy-amopyroquine; DH-ApQ)*: A mixture of 3-(pyrrolidin-1-ylmethyl)aniline (70 mg, 0.40 mmol), 4,7-dichloroquinoline (79 mg, 0.40 mmol), acetonitrile (1 mL), water (1 mL), and 12 M HCl (33 µL, 0.40 mmol) was heated in a sealed tube at 110 °C for 4 h. The reaction was cooled to r.t., and 5 M NaOH (160 µL, 0.79 mmol) was added to the stirred solution. The product was collected by filtration and ½ of this crude material was purified by column chromatography on silica (10 g, 0–100% ethylacetate in iso-hexane as eluent) to generate 7-chloro-N-[3-(pyrrolidin-1-ylmethyl)phenyl]quinolin-4-amine (25 mg, yield 19%). LCMS [M + H] + *m/z* 338; 1H NMR (400 MHz, DMSO-d6) ppm 1.64–1.74 (m, 4H), 2.39–2.48 (m, 4H), 3.59 (s, 2H), 6.92 (d, *J* = 5.4 Hz, 1H), 7.06–7.11 (m, 1H), 7.22–7.27 (m, 1H), 7.29–7.34 (m, 1H), 7.35 (t, *J* = 7.4 Hz, 1H), 7.56 (dd, *J* = 9.0, 2.2 Hz, 1H), 7.89 (d, *J* = 2.2 Hz, 1H), 8.43 (d, *J* = 9.0 Hz, 1H), 8.46 (d, *J* = 5.4 Hz, 1H), 9.09 (s, 1H).

## Results

### AQ inhibits rRNA transcription and induces degradation of the catalytic subunit of Pol I

**Fig. 1:**
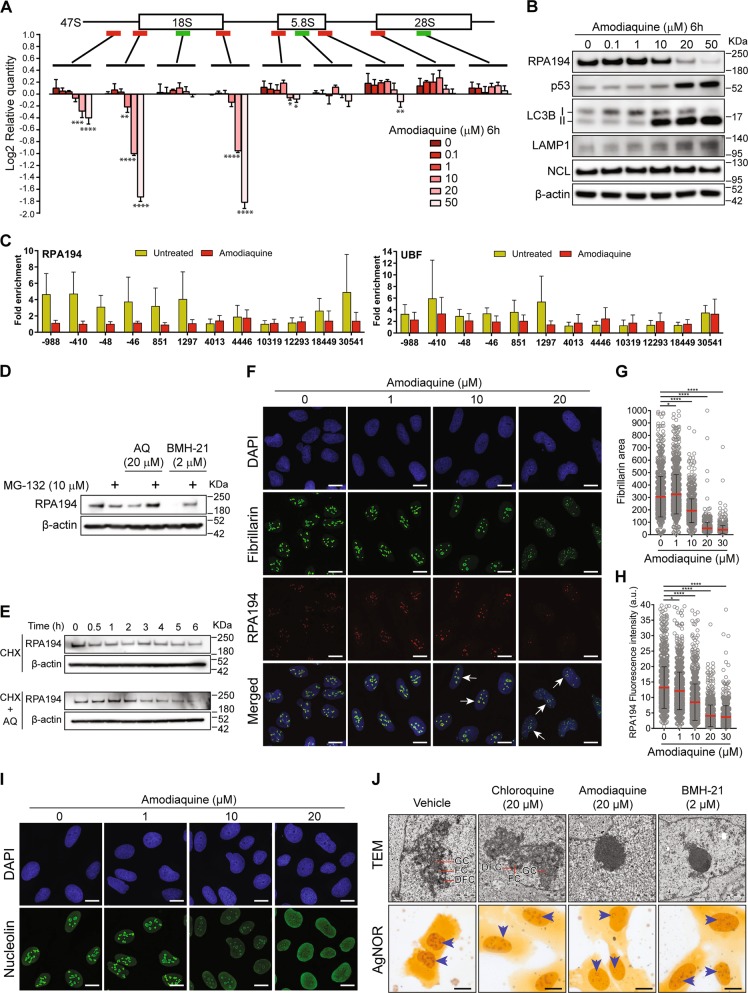
Amodiaquine inhibits ribosome biogenesis. **a** RT-qPCR analysis of 47S rRNA precursor demonstrated a reduction of 47S transcription in U2OS cells treated with increasing concentrations of AQ. Red bars: 47S-specific primers; Green bars: regions shared with mature rRNAs. Data shown as mean ± SD of triplicate wells and are representative of triplicate treatments; Statistical significance was calculated by one-way ANOVA using log-transformed data and Dunnett’s multiple test comparison (*p < 0.05, **p < 0.01, ***p < 0.001, ****p < 0.001). **b** Immunoblot analysis of U2O2 cells treated as in **a** shows reduction of RPA194 protein, along with p53 activation and LC3-II and LAMP1 accumulation in U2OS cells treated with increasing doses of AQ during 6 h. Nucleolin (NCL) levels show no change. **c** Chip-qPCR analyses revealed a reduction of RPA194 binding to rDNA sequence, but UBF binding was less affected. U2OS cells treated with 20 µM of AQ during 6 h (*n* = 3). Data shown as mean ± SD of triplicate wells and are representative of three independent experiments. **d** Degradation of RPA194 induced by AQ is proteasome dependent. U2OS cells were treated with proteasome inhibitor MG-132 during 1 h before addition of AQ and BMH-21 during 6 h. **e** U2OS cells were incubated with cyclohexamide (CHX) in the absence and presence of AQ during 6 h. **f** Immunofluorescence analysis showed a decrease in RPA194 signal and disruption of fibrillarin’s localization pattern. U2OS cells treated with 20 µM of AQ during 6 h. Nuclear DNA was counterstained with DAPI. White arrows indicate nucleolar caps. Scale bars, 10 µm. **g**, **h** Quantification of fibrillarin area and RPA194 intensity in >800 cells per condition, treated as in **f**. Data shown as mean ± SD and is representative of two independent experiments; Statistical significance was calculated by one-way ANOVA using Kruskal–Wallis test and Dunn’s multiple test comparison (*p < 0.05, **p < 0.01, ***p < 0.001, ****p < 0.001). **i** Nucleolin is translocated into the nucleoplasm. Nuclear DNA was counterstained with DAPI. U2OS cells treated with increasing doses of AQ during 6 h. Scale bars, 10 µm **j** U2OS cells were treated with CQ, AQ and BHM-21 during 6 h and fixed for transmission electron microscopy (TEM) for nucleoli analysis. Scale bars, 1 µm; GC granular component, FC fibrillar center, DFC dense fibrillar component. AgNOR staining was used to identify changes in nucleolar organizer regions. Blue arrows indicate nucleolus. Scale bars, 10 µm

While searching for Pol I inhibitors among FDA-approved drugs, we assessed the impact of AQ on levels of 47S rRNA, using a set of primers targeting mature 18S, 5.8S and 28S rRNAs and six primers for regions present exclusively in the 47S precursor rRNA. Precursor-specific regions showed a dose-dependent decrease in 47S synthesis (Fig. 1a; red bars), without affecting the levels of mature ribosomes (Fig. 1a; green bars). Furthermore, we observed a dose-dependent degradation of the RPA194 subunit of Pol I and p53 accumulation (Fig. 1b). As expected, AQ also induced accumulation of autophagosome marker LC3-II and lysosomal marker LAMP1 (Fig. 1b) [18]. To explore the transcriptional status of the rDNA, we analyzed the effect of AQ on UBF and RPA194 association with rDNA using chromatin immunoprecipitation (ChiP). UBF is essential for the formation of the PIC and further recruitment of the Pol I complex to the rDNA [24]. After AQ exposure, RPA194 dissociated from the rDNA (Fig. 1c), particularly the promoter region (positions −48 and −46), upstream (positions −988 and −410) and the 5′ETS region (positions 851 and 1297). In contrast, UBF was only slightly affected (Fig. 1c).

We hypothesized that the mechanism by which AQ alters ribosome biogenesis may resemble the effects of BMH-21, an inhibitor of ribosome biogenesis that also triggers a ubiquitin-proteasome-dependent degradation of RPA194 [25]. Indeed, proteasome inhibition partially rescued the levels of RPA194 upon treatment with AQ (Fig. 1d). The protein translation inhibitor cycloheximide caused reduction in RPA194 levels after 3 h of treatment (Fig. 1e). We assessed the transcriptional status of genes which products compose the complex. Both AQ and BMH-21 induced a significant downregulation of *POLR1D, POLR1E,* and *TAF1B* components of the Pol I PIC. BMH-21 downregulated *UBFT, TWISTNB*, and *POLR1A* and upregulation of *POLR1B, POLR1C, TAF1C,* and *TAF1D* (Supplementary Fig. 1).

Under nucleolar stress conditions [26, 27], areas of rDNA repeats called nucleolar caps are reorganized toward the nucleolar periphery. These can be induced either by DNA damage or compounds with affinity for GC rich regions of DNA such as actinomycin D [28] and BMH-21 [12]. To assess changes in nucleolar structure upon AQ exposure, we used immunofluorescence for proteins located in the three nucleolar compartments: RPA194 located at the fibrillar center and fibrillarin in the dense fibrillary component (DFC) both translocate to nucleolar caps, while nucleolin, mainly located at the granular component, translocates to the nucleoplasm under stress [29]. An AQ-dose-dependent dismantling of the nucleolus was observed as indicated by the reduction of fibrillarin area (Fig. 1f, g), and decreased RPA194 intensity (Fig. 1f, h) along with the generation of nucleolar caps (Fig. 1f, white arrows) and nucleolin translocation into the nucleoplasm (Fig. 1i), without altering its total level (Fig. 1a). Moreover, AQ also induced translocation into the nucleoplasm of the nucleolar helicase DDX21 (Supplementary Fig. 2), an enzyme involved in the synthesis and processing of rRNA [30].

We then asked whether the nucleolar changes observed for AQ: (i) can be reproduced by its analog CQ; and (ii) are comparable with those induced by BMH-21. For this, we employed electron microscopy in order to detect subtle changes. AQ induced dramatic condensation of the nucleolar chromatin along with loss of the distinctive nucleolar regions FC, DFC, and GC, similarly to BMH-21(Fig. 1j and Supplementary Fig. 3). In contrast, CQ did not affect chromatin and the nucleolar subcompartments remained preserved (Fig. 1j), consistent with AgNOR staining that detects argyrophilic proteins located at the FC/DFC regions [31]. AQ and BHM-21, but not CQ, induced a collapse of the FC/DFC regions toward nucleolar borders detected as ring-shaped structures (Fig. 1j). These results showed extensive inhibition of rRNA synthesis and nucleolar remodeling induced by AQ.

### AQ stabilizes p53 via the RPL5/RPL11-5S rRNA checkpoint, independent of ATM

**Fig. 2:**
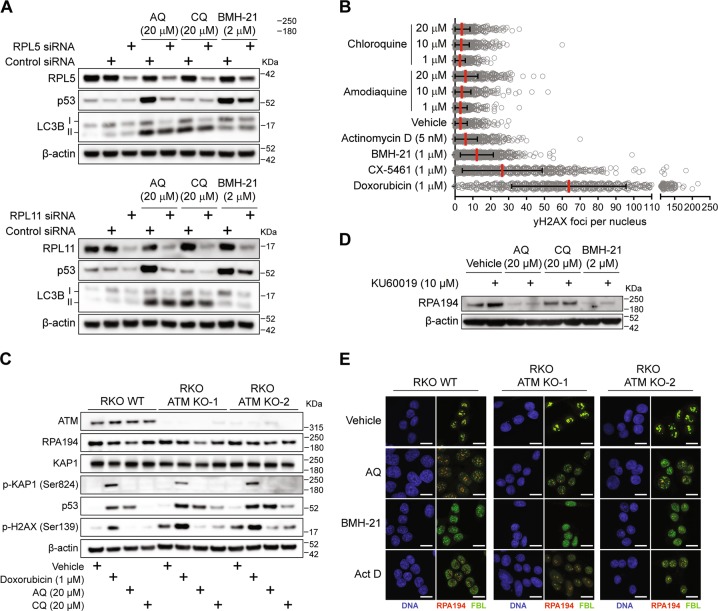
Amodiaquine stabilizes p53 through RPL5/RPL11 nucleolar checkpoint and does not require ATM activation. **a** Knockdown of RPL5 and RPL11 prevent p53 activation after exposure to AQ. Cells were treated with siRNA against RPL5 and RPL11 for 24 h. After, cells were incubated with AQ, CQ, and BHM-21 during an additional 6 h. β-actin levels were used as loading control. **b** Quantification of γ-H2AX positive foci number per nucleus after 24 h of incubation with CQ, AQ, actinomycin D, BMH-21, CX-5461 and doxorubicin (>1000 cells per group). Data shown as mean ± SD of duplicate wells and are representative of two independent experiments. (**c**) ATM is not required for RPA194 degradation and p53 activation induced by AQ. Immunoblot analysis for ATM, RPA194, KAP1, p-KAP1, p53, p-H2AX, and β-actin in WT RKO colon cancer cell line and two ATM knockout clones. Cells were incubated with doxorubicin, AQ, and CQ during 6 h. **d** ATM inhibition does not prevent RPA194 degradation. U2OS cells were pretreated with the ATM inhibitor KU60019 during 1 h and then incubated with AQ, CQ, and BMH-21 during 6 h. **e** Nucleolar caps induced by AQ do not require ATM. Immunofluorescence staining of RPA194 and fibrillarin in WT RKO colon cancer cell line and two ATM knockout clones treated as in **c**. Scale bars, 10 µm

AQ is more efficient than CQ in stabilizing p53 at equimolar concentrations [18, 19], however, the mechanism underlying this difference is unknown. Ribosome biogenesis stress leads to p53 activation mainly through the interaction between the RPL5/RPL11/5S rRNA complex and MDM2, inhibiting MDM2’s ubiquitin ligase activity and thereby stabilizing/activating p53 [32]. To examine whether this checkpoint is triggered by AQ, we depleted the nuclear pools of RPL5 and RPL11 using siRNA knockdown. After AQ exposure, stabilization of p53 was reduced to control levels when knocking down either RPL5 or RPL11 (Fig. 2a), indicating that the AQ-induced p53 activation reflects ribosome biogenesis stress. Consistently, p53 activation induced by BHM-21 was partially reversed by RPL5/11 knockdown, while p53 remained unaffected upon CQ treatment (Fig. 2a). Thus, the RPL5/RPL11/5S rRNA checkpoint mediates the AQ-induced p53 stabilization.

Transcription of rDNA becomes inhibited upon DNA damage by ATM kinase-mediated signaling [33, 34]. We observed that AQ is capable of intercalating DNA depending on GC content, analogous to BMH-21 (Supplementary Fig. 4). To assess potential DNA damaging effects, we measured γ-H2AX foci formation in U2OS cells exposed to diverse compounds for 24 h, compared with the topoisomerase II inhibitor and DNA intercalator doxorubicin as a positive control [35]. Both AQ and CQ produced similar amounts of foci comparable with actinomycin D, while the foci levels induced by BMH-21 were slightly higher (Fig. 2b), yet still lower than the γ-H2AX foci induced by CX-5461. Indeed, both BMH-21 and CX-5461 inhibit rRNA synthesis; however, they differ in their genotoxic capacity. Whereas BMH-21 inhibits rDNA transcription without significant DNA damage [11], CX-5461 causes DNA damage and activates ATM/ATR signaling [9, 10]. To investigate any role of ATM in AQ-dependent impact on RPA194 and p53 levels, we exposed ATM-knockout cells to AQ, CQ, and doxorubicin. AQ-induced RPA194 degradation and p53 stabilization were evidenced even in the absence of ATM (Fig. 2c). Doxorubicin does not induce RPA194 degradation; however, it triggers p53 stabilization as well as phosphorylation of the DNA damage markers KAP1 and H2AX (Fig. 2c). As doxorubicin also partly inhibits rRNA synthesis [36], yet does not induce RPA194 degradation, DNA damage is not directly linked to RPA194 degradation. Consistently, preincubation with the ATM inhibitor KU60019 did not rescue the RPA194 degradation induced by AQ and BMH-21 (Fig. 2d). Upon induction of double-strand breaks in the rDNA, ATM signaling leads to generation of nucleolar caps and chromatin remodeling. However, chemically induced nucleolar caps are not prevented by ATM inhibition [37]. Similarly, we observed that nucleolar caps are induced by AQ at similar levels regardless of ATM status, analogous to caps induced by BMH-21 and actinomycin D (Fig. 2f). Taken together, these results show that RPA194 degradation, p53 stabilization and nucleolar caps induced by AQ occur independently of ATM signaling.

### AQ induces ribosome biogenesis stress independently of its autophagy-inhibitory activity

**Fig. 3:**
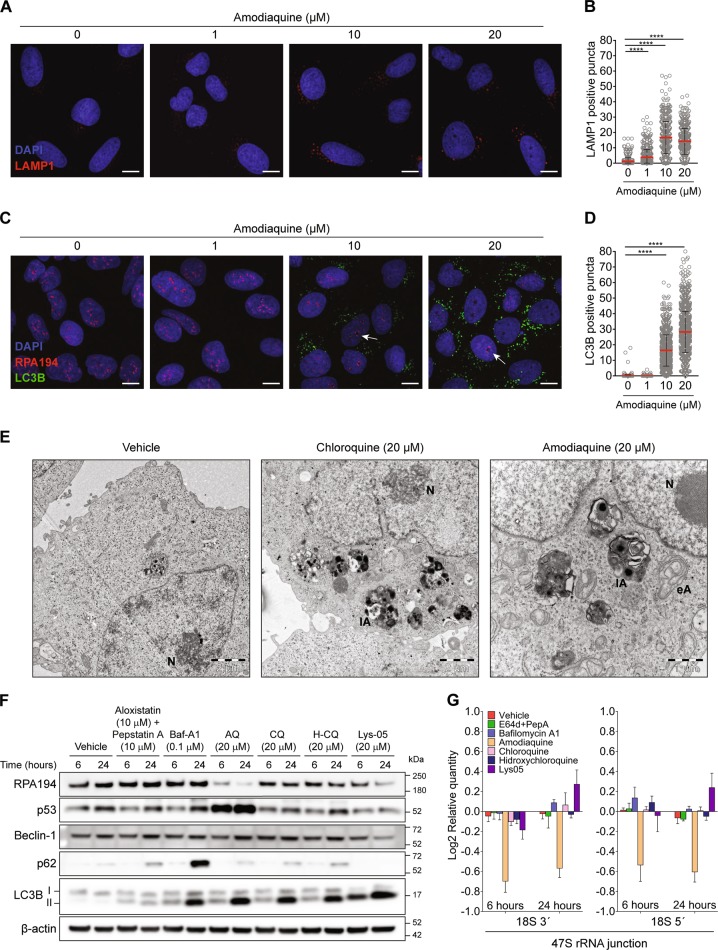
Ribosome biogenesis stress induced by amodiaquine is not related to its autophagy inhibition activity. **a** AQ induces accumulation of LAMP1-positive and **c** LC3-positive puncta along with RPA194 positive nucleolar caps. U2OS cells were treated with increasing doses of AQ during 6 h and stained. White arrows indicates nucleolar caps. Scale bars, 5 µm. **b** Quantification of LAMP1-positive and **d** LC3B-positive puncta in cells treated as in **a** and **c**. Statistical significance was calculated by one-way ANOVA using Kruskal–Wallis test and Dunn’s multiple test comparison (*p < 0.05, **p < 0.01, ***p < 0.001, ****p < 0.001). **e** Ultrastructural analysis shows accumulation of cytoplasmic vesicles along with alteration of nucleolar structure induced by AQ. Transmission electron microscopy of U2OS cells treated during 6 h with CQ and AQ. N nucleolus; eA early autophagosome; lA late autophagosome. **f** Comparison between autophagy inhibitors shows that only AQ induces degradation of RPA194 and activation of p53. Immunoblot analysis of RPA194, p53, beclin-1, p62, LC3B, and β-actin in U2OS cells treated with aloxistatin + pepstatin A, bafilomycin-A1 (Baf-A1), amodiaquine (AQ), chloroquine (CQ), hidroxychloroquine (H-CQ) and Lys-05 during 6 and 24 h. **g** RT-qPCR analysis of 18S 3′and 18S 5′ junction of the 47S rRNA precursor in cells treated as in **f**. Data shown as mean ± SD of triplicate wells and are representative of three independent experiments

AQ, along with other members of the 4-aminoquinoline family, including CQ, hydroxychloroquine (H-CQ), and Lys-05 (dimeric CQ) inhibit autophagy [17]. While 4-aminoquinolines were thought to inhibit autophagy by increasing lysosomal pH [17], recent evidence challenges this idea [38]. Cells treated with 1–10 μM AQ for 6 h accumulated lysosomes (LAMP1-positive puncta) (Fig. 3a, b) while concentrations between 10 and 20 μM further enhanced autophagosome accumulation (Fig. 3c, d; LC3B puncta). Notably, 10–20 μM AQ also induced generation of nucleolar caps (Fig. 3c), showing that both autophagy inhibition and nucleolar stress take place simultaneously. Strikingly, even though AQ and CQ showed similar levels of lipidated LC3 (LC3-II) (Fig. 2a) and accumulation of cytoplasmic vesicles (Fig. 3e), only AQ caused remarkable condensation of nucleolar chromatin (Fig. 3e). Therefore, we asked whether autophagy inhibition contributes to the AQ-induced nucleolar stress and how does this relate to other 4-aminoquinoline family members and other mechanisms of autophagy inhibition. To elucidate these issues, we treated U2OS cells with cathepsin inhibitors pepstatin A and aloxistatin (a.k.a E64d), that prevent autophagy through suppression of protein cleavage inside lysosomes; and bafilomycin-A1, an inhibitor of the vacuolar-type H^+^-ATPase that maintains acidic pH inside the lysosomes. Increase in pH inhibits hydrolases and autophagy [17]. Although treatments with aloxistatin/pepstatin A and bafilomycin-A1 induce accumulation of LC3-II and prevent p62 degradation, no effect was observed on either RPA194 protein levels or p53 stabilization, in contrast to effects of AQ, at 6 and 24 h of treatment (Fig. 3f). Among the 4-aminoquinoline family members, CQ and H-CQ inhibit autophagy with efficiency similar to AQ, but RPA194 degradation and p53 stabilization was only induced by AQ (Fig. 3f). At 6 h, Lys-05 induced the strongest LC3-II accumulation compared with AQ, CQ, and H-CQ (Fig. 3f), consistently with previous results [39]. However, at 6 h the effect of Lys-05 on RPA194 degradation was subtle and there was no p53 stabilization. Furthermore, only AQ induced a strong reduction of 47S rRNA synthesis among all autophagy inhibitors tested (Fig. 3g). Overall, these findings indicate that ribosome biogenesis stress is not a general consequence of autophagy inhibition and that AQ stands out among the 4-aminoquinoline family as a compound operating through two independent mechanisms: autophagy inhibition in the cytoplasm and ribosome biogenesis stress in the nucleolus.

### AQ perturbs transcription analogous to CQ and BMH-21

**Fig. 4:**
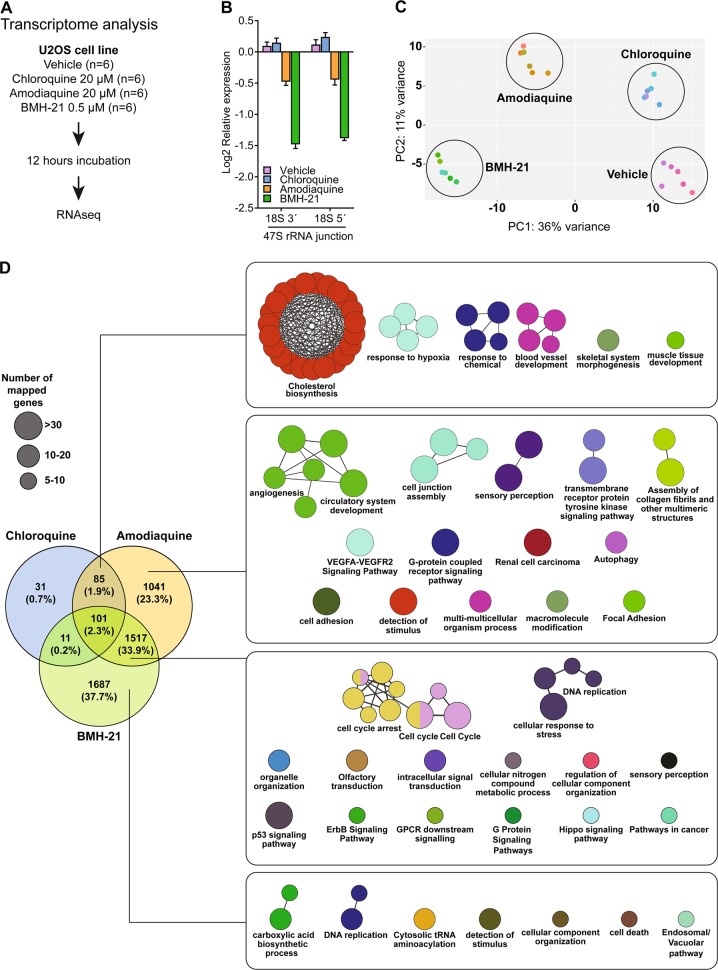
Transcriptional perturbation induced by Amodiaquine resembles that of Chloroquine and BMH-21. **a** Experimental design for RNAseq analysis. **b** AQ reduces the synthesis of 47S with less intensity than BMH-21. RT-qPCR analysis for the 18S 3′ and 5′ junctions in the 47S rRNA transcript for cells treated as in **a**. Data shown as mean ± SD of three treatments. **c** Principal component analysis based on RNAseq data, color-coded according to each treatment. The scatter plot shows the position of samples based on the two first principal components. **d** Gene ontology and pathway analysis comparing common and unique features among differentially expressed genes disrupted by AQ, CQ, and BMH-21. ClueGO integrates GO terms and pathways to create a functionally organized network. Node size represents amount of mapped genes and node color is assigned randomly. Nodes with two colors contains genes that converged from different databases

Transcriptome analysis can identify shared and unique mechanisms of action among compounds, pointing to links between biological activities and diseases [40]. Unlike CQ, AQ triggers ribosome biogenesis stress, reminiscent of mechanistic features of BMH-21. To elucidate the transcriptional networks underlying these features, we performed a transcriptome analysis using RNAseq on U2OS cells treated with AQ, CQ, and BMH-21 (Fig. 4a). BMH-21 inhibited rRNA synthesis at lower concentrations than AQ (Fig. 4b), while CQ did not impact the 47S rRNA level (Fig. 4b). Principal component analysis showed that AQ segregated separately from CQ and BMH-21, suggesting unique properties (Fig. 4c). To gain further mechanistic insights, we performed a differential expression analysis between each compound and the vehicle using the DEseq algorithm, applying as cut-off a Bonferroni corrected *p* value < 0.001 and gene expression fold changes >0.6 and <−0.6. Under equimolar concentrations, AQ induced more gene expression changes than CQ (2774 vs 228 genes, respectively). Notably, BMH-21 induced the most dramatic perturbation, with 3316 affected genes. Differentially expressed genes were further sorted among the three compounds using Venn diagrams to identify common and unique gene perturbations.

Among the CQ-regulated genes, 37% (85 out of 228) were also regulated by AQ, including transcripts enriched for cholesterol biosynthesis, response to hypoxia and blood vessel development (Fig. 4d). Indeed, CQ impairs cholesterol uptake and reduces processing of extracellularly derived cholesterol esters, rendering cells dependent on the cholesterol biosynthesis [41]. The impact of CQ on vessel normalization reflects lysosomal dysfunction and sustained activation of Notch1. The latter, autophagy-independent mechanism, seems to underlie the antineoplastic effect of CQ in vivo [42]. Interestingly, we observed that AQ and CQ induce expression of the endoplasmic reticulum (ER) stress-related genes GADD34 (PPP1R15A), CHOP (DDIT3), REDD1 (DDIT4), BNIP3 and BNIP3L (Nix). In a more detailed biochemical analysis of ER stress, we observed moderate activation of ATF4, together with phosphorylation of PERK and eIF2α, indicating activation of the PERK branch of the unfolded protein response [43], in the absence of ATF6 cleavage and lack of translation of spliced XBP1 (Supplementary Fig. 5). The transcriptional overlap between AQ and CQ suggests that AQ shares most of the autophagy/lysosome-disrupting mechanisms with CQ.

Furthermore, 1041 genes were specifically affected by AQ, enriched in angiogenesis/VEGF, autophagy and cell adhesion pathways. Among all genes perturbed by the three compounds, 33.9% overlapped between AQ and BMH-21, including those related to cell-cycle arrest, DNA replication stress, and p53 signaling, consistent with our results and previous studies [18, 25]. Among the p53-regulated genes we observed higher expression of the cyclin dependent kinase inhibitor CDKN1A (p21) and the proapoptotic genes PMAIP1 (Noxa) and BBC3 (Puma). Upon siRNA mediated knockdown of p53, the AQ-induced increase in expression of these genes was decreased, often to a degree that was no longer significant compared with vehicle-treated cells (Supplementary Fig. 6). These results implicate the p53-mediated checkpoint signaling in response to AQ-induced ribosome biogenesis stress as a significant contributor to the observed cellular transcriptional responses with likely impact toward cell-cycle inhibition and cell death machineries (Supplementary Fig. 6). BMH-21 alone perturbed the expression specifically of 1687 genes, implicated in carboxylic acid synthesis and downregulating components of tRNA aminoacylation, including AARS, CARS, EPRS, GARS, IARS WARS, YARS, and VARS. A total of 101 perturbed genes (2.3%) were shared by all three compounds.

These results reveal that AQ induces transcriptional changes that overlap with those induced specifically either by CQ or BMH-21, thereby overall consistent with the concept that AQ impacts cells simultaneously through two mechanisms of action: autophagy inhibition and ribosome biogenesis stress.

### AQ kills CRC cells

**Fig. 5:**
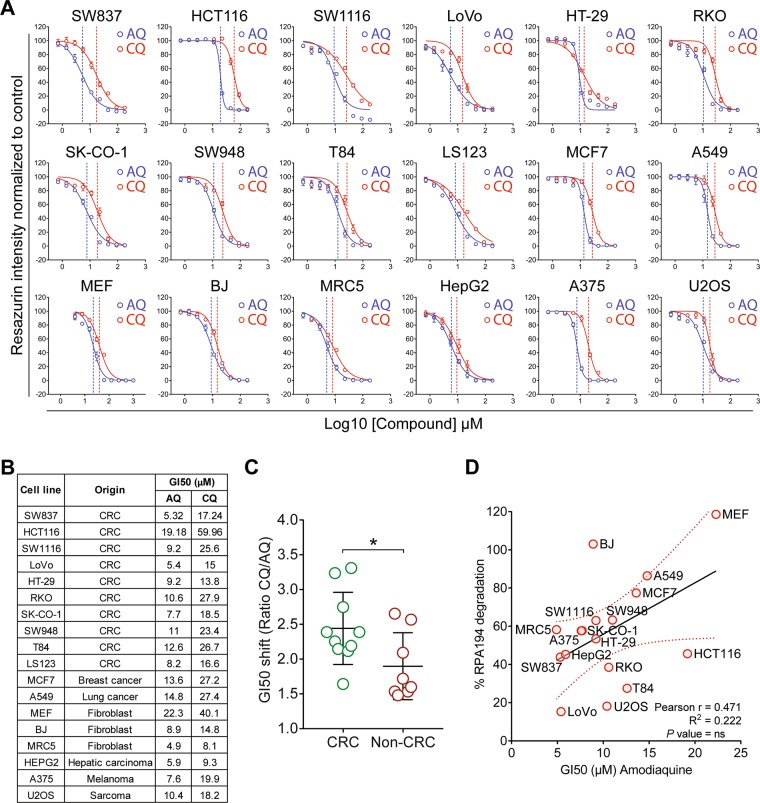
Amodiaquine is more cytotoxic than chloroquine, particularly in colorectal cancer cells **a** Dose responses curves and **b** growth-inhibitory concentrations (GI50s) of AQ and CQ in a panel of colorectal (CRC) and non-CRC cell lines. Cells were incubated with compounds for 72 h. Fitted dose response curves display mean and standard deviation from three experiments. In **a**, dashed vertical lines represent the growth-inhibitory concentrations (GI50). **c** CRC cell lines are more sensitive to AQ than non-CRC lines. Comparison of GI50 shift between AQ and CQ in CRC and non-CRC cell lines using CQ/AQ ratios calculated from triplicate dose response curves. Ratios were analyzed using unpaired *t* test; *P value < 0.05. **d** Correlation between GI50s obtained after 72 h and RPA194 degradation obtained by quantitative immunoblot after 6 h of incubation with AQ

AQ is more cytotoxic against cancer cells than CQ [18, 44], albeit AQ displays similar or lower autophagy/lysosome inhibition [18, 44]. Unlike for AQ, the CQ-induced cell death depends on glucose [44]. These differences suggest that the unique impact of AQ on rDNA transcription may explain its higher cytotoxicity. Recently, ribosome biogenesis was highlighted as a clinically relevant target in CRC [13]. While testing toxicity in cell culture, the growth-inhibitory concentrations (GI50s) of AQ were consistently lower than for CQ in a panel of 18 human cancer and immortalized cell lines (Fig. 5a, b). The CQ:AQ GI50s ratios were higher among the CRC cell lines, supporting higher sensitivity to AQ (Fig. 5c). When correlating the sensitivities among all cell lines (GI50s) with the extent of AQ-induced RPA194 degradation as a marker for ribosome biogenesis stress, a *R*^2^ of 0.22 was found (Fig. 5d). Such partial correlation implies that RPA194 degradation per se cannot serve as a universal predictor of AQ-induced cell death under this type of nucleolar stress, the overall outcome of which likely reflects also additional factors such as the p53 status or adaptive pro-survival pathways that impact the fate of AQ-treated cells. Notably, when RPA194 degradation was analyzed in nontransformed human cell types, we observed either only partial degradation (IMR-90) or complete lack of RPA194 degradation (BJ fibroblasts and HEKa primary keratinocytes), overall indicative of resistance to AQ compared with cancer cells (Supplementary Fig. 7).

### The reactive metabolite of AQ is not involved in nucleolar activity

**Fig. 6:**
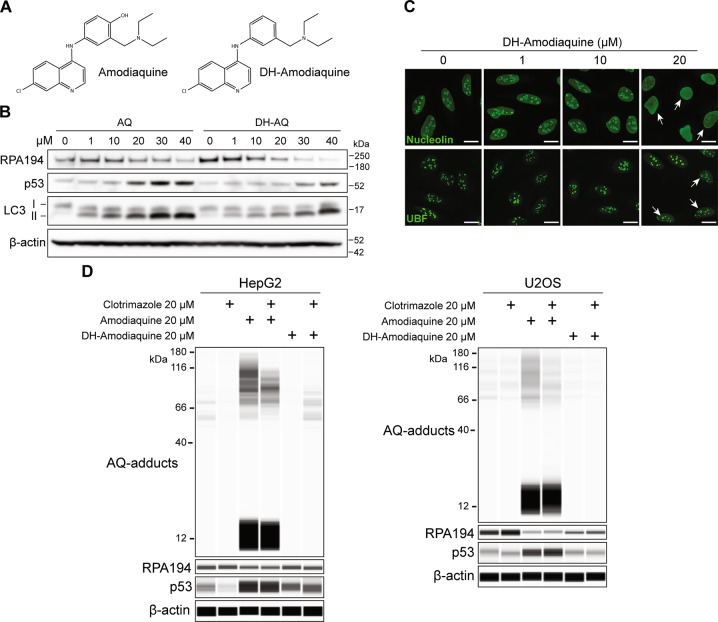
AQ-induced protein adduction is not involved in nucleolar stress **a** Chemical structures of amodiaquine and its analog without the reactive group, deshydroxy-amodiaquine (DH-AQ). **b** Immunoblot analysis of RPA194, p53 and LC3 in U2OS cells treated with increasing doses of AQ and its nonreactive analog DH-AQ during 6 h. **c** Nucleolin translocation and UBF-positive nucleolar (white arrows) caps were detected by immunofluorescence in U2OS cells treated as in **a**. Scale bars, 10 µm. **d** Capillary immunoblot of HepG2 and U2OS cells treated with 20 µM of AQ and DH-AQ during 6 h. Cells were treated with 20 µM of clotrimazole for 1 h prior to adding AQ and DH-AQ, maintaining the same concentration of clotrimazole for the next 6 h of incubation. AQ-adducts were detected using a monoclonal antibody against AQ

AQ combined with artesunate is indicated by the WHO for the treatment of uncomplicated *Plasmodium falciparum* and *Plasmodium vivax* infections and seasonal malaria chemoprevention [45], while AQ treatment for malaria prophylaxis declined during the 80s following reports of idiosyncratic hepatotoxicity and agranulocytosis [46]. The precise origin of these idiosyncratic reactions is not yet understood. AQ contains a p-aminophenol moiety and undergoes extensive bioactivation with formation of a reactive quinoneimine intermediate that strongly depends on cytochrome P450 activity in the liver [47] and myeloperoxidase in neutrophils [48]. This reactive metabolite can bind proteins covalently, generating protein adducts that are suspected to be responsible for the toxicity [18, 49]. To assess whether the reactive group in the AQ metabolite contributes to the nucleolar impairment, we synthesized an AQ analog removing the hydroxyl group to disrupt the aminophenol moiety (deshydroxy-amodiaquine, DH-AQ; Fig. 6a). The DH-AQ still caused p53 activation, RPA194 degradation (Fig. 6b), nucleolar cap formation and dissipation of nucleolin, only slightly lower than AQ (Fig. 6c). This implies that the reactive metabolite does not determine the nucleolar stress, since the (–OH) group does not robustly affect the nucleolar impact of AQ. However, by modulating the electrochemical behavior of the compound, the (-OH) may improve interactions with rDNA and/or RPA194.

The CYP450 isoform CYP2C8 promotes generation of N-desethylamodiaquine (DE-AQ) [50], the main stable metabolite of AQ, responsible for the antimalarial activity. We observed that DE-AQ retains AQ’s autophagy- and ribosome biogenesis-inhibitory activities (Supplementary Fig. 8). Furthermore, the cytochromes CYP2C8, CYP2C9, CYP2D6, and CYP3A4 contribute to bioactivation of AQ into protein-reactive quinoneimines [51]. The human hepatoma cell line HepG2, a proposed model for hepatic toxicity, shows high expression and activity of cytochromes [52]. Compared with U2OS, the HepG2 cells expressed higher mRNA levels of all cytochrome genes we tested, particularly of CYP3A4. HepG2 cell line is affected by DH-AQ in a similar way as U2OS, inducing p53 activation and RPA194 degradation (Supplementary Fig. 9). Capillary immunoblotting using an anti-AQ antibody detected AQ-induced protein adduction in both HepG2 and U2OS cells, generating a smear between ~60 and ~120 kDa, with a stronger signal for HepG2 (Fig. 6d), and an intense signal around ~12 kDa (Fig. 6d), probably corresponding to AQ-glutathione adducts, the detoxification product catalyzed by glutathione S-transferases. This reaction is observed for AQ and reactive metabolites derived from other drugs [53]. Importantly, the absence of the reactive group in DH-AQ completely abrogated the formation of protein adducts, however, DH-AQ still triggered RPA194 degradation in U2OS cells and weaker p53 activation in both cell lines. (Fig. 6d). In addition, in order to inhibit cytochrome activity, cells were treated with the potent nonselective cytochrome inhibitor clotrimazole, which has been shown to inhibit CYP2C8, CYP2C9, CYP2D6, and CYP3A4 [54, 55]. In both cell lines, the treatment with clotrimazole reduced the levels of AQ-protein adducts, however, in HepG2, DH-AQ is still capable of slightly decreasing RPA194 levels and activating p53 while in U2OS, DH-AQ induces the same level of degradation of RPA194, showing that cytochrome activity is not required for the nucleolar effects induced by AQ.

These results provide an additional mechanistic feature of AQ, showing that the nucleolar activity is retained in the absence of the reactive intermediates, and supporting feasibility of chemical optimization of AQ to enhance the nucleolar disruption capacity while eliminating idiosyncratic side effects.

### An AQ-derived analog that inhibits both autophagy and ribosome biogenesis

**Fig. 7:**
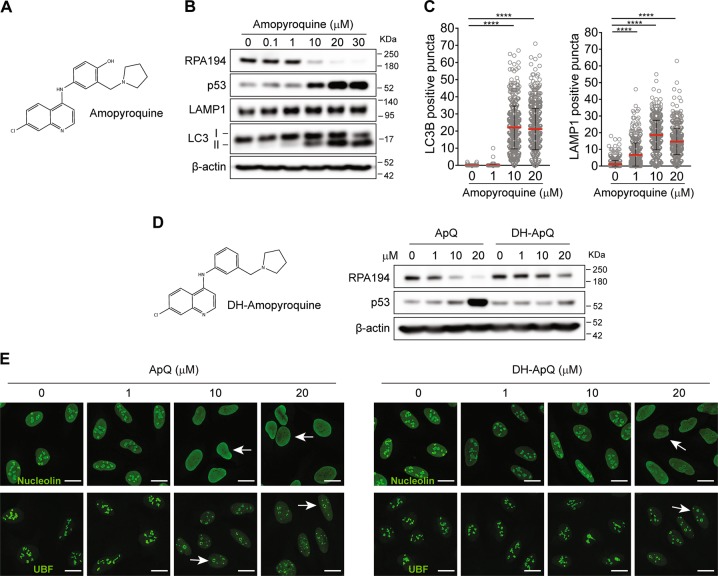
Amopyroquine exerts nucleolar stress in a similar way as its analog amodiaquine **a** Chemical structure of amopyroquine (ApQ). **b** ApQ induces similar alterations to those of AQ. Immunoblot analysis of RPA194, p53, LC3, LAMP1, and nucleolin in U2OS cells treated with increasing doses of AQ during 6 h. **c** Quantification of LC3B and LAMP1-positive puncta in cells treated as in **b**. **d** ApQ analog without the reactive group (deshydroxy-amopyroquine; DH-ApQ) shows somewhat less activation of p53 and less RPA194 degradation. Immunoblot analysis of RPA194 and p53 in U2OS cells treated with increasing doses of ApQ and its analog DH-ApQ during 6 h. **e** DH-ApQ has less activity than ApQ inducing nucleolin translocation and generation of UBF-positive nucleolar. Immunofluorescence analysis of U2OS cells treated as in **d**. White arrows indicate nucleolin translocation into nucleoplasm and UBF-positive nucleolar caps. Scale bars, 10 µm

To investigate whether chemical optimization of AQ could increase its efficiency of rDNA transcription inhibition, we first tested amopyroquine (ApQ), a commercially available structural analog of AQ. In ApQ, the 2-diethylamino side chain is replaced by a cyclic pyrrolidone one (Fig. 7a). Notably, ApQ was more potent than AQ in inducing RPA194 degradation and p53 stabilization, with comparable amounts of LC3-II generation (Fig. 7b) and similar accumulation of LC3 and LAMP1 puncta (Fig. 7c). ApQ contains the p-aminophenol moiety that could potentially generate reactive intermediates. We chemically modified ApQ by removing the hydroxyl group (deshydroxy-amopyroquine DH-ApQ; Fig. 7d). Whereas DH-ApQ still triggered nucleolar stress, nucleolin dissipation and UBF-positive nucleolar caps (Fig. 7e), the extent of RPA194 degradation and p53 stabilization were slightly affected (Fig. 7d). These findings showed that side chain-modified AQ analogs may preserve or even increase the nucleolar disruption capacity, thereby supporting the notion that optimized analogs deserve future exploration as candidate anticancer compounds.

## Discussion

Our findings show that the FDA-approved antimalarial drug AQ inhibits ribosome biogenesis, an activity not shared by other 4-aminoquinoline family members. AQ triggers proteasome-dependent degradation of RPA194 (POLR1A), a feature reported for the experimental drugs BMH-21 [12] and BMH-22 [25, 56]. Notably, this is the first example of a FDA-approved drug capable of triggering Pol I degradation.

Targeting ribosome biogenesis has been proposed as a therapeutic strategy for hematological malignancies [57], ovarian [58], prostate [59], and MYC-driven cancers [60]. Relevance to CRC has been highlighted by the discovery that oxaliplatin, used as standard-of-care for CRC patients, kills cancer cells mainly due to its capacity to impair ribosome synthesis [13]. In this context, the identification of molecular subtypes with greater sensitivity to ribosomal stress will enable the selection of patients who might benefit from the use of ribosome biogenesis inhibitors [1].

Ongoing clinical trials are addressing the potential of CQ and H-CQ in combinatorial treatments for various cancer types. Most such trials were designed with the rationale to increase the efficacy of other anticancer therapies through inhibition of treatment-induced autophagy. Although it is too early to draw any conclusions, data from first clinical trials and additional preclinical data suggest a potential for implementing these drugs in anticancer treatment [61]. To the best of our knowledge, no cancer clinical trial has yet been reported for AQ. Our findings show that while AQ and CQ share similar autophagy-inhibitory activity, AQ also displays an additional, potent mechanism to restrain cancer cell growth. Thus, the dual activity of AQ in the cytoplasm and the nucleolus makes this drug a promising candidate for cancer therapy.

In conclusion, we have demonstrated that AQ inhibits ribosome biogenesis and disrupts nucleolar structure, triggering degradation of RNA polymerase I. Moreover, AQ exerts high cytotoxic activity against CRC, a neoplasia with predicted sensitivity to ribosome biogenesis inhibitors. Our study supports the rationale for repurposing an old and inexpensive drug to target an emerging vulnerability in cancer, which may translate into more affordable and accessible therapies.

## Supplementary information

Supplementary material

## References

[1] Pelletier J, Thomas G, Volarevic S (2018). Ribosome biogenesis in cancer: new players and therapeutic avenues. Nat Rev Cancer.

[2] Wang M, Lemos B (2017). Ribosomal DNA copy number amplification and loss in human cancers is linked to tumor genetic context, nucleolus activity, and proliferation. PLoS Genet.

[3] Derenzini M, Montanaro L, Trere D (2009). What the nucleolus says to a tumour pathologist. Histopathology.

[4] Bywater MJ, Poortinga G, Sanij E, Hein N, Peck A, Cullinane C (2012). Inhibition of RNA polymerase I as a therapeutic strategy to promote cancer-specific activation of p53. Cancer Cell.

[5] Zhao J, Yuan X, Frodin M, Grummt I (2003). ERK-dependent phosphorylation of the transcription initiation factor TIF-IA is required for RNA polymerase I transcription and cell growth. Mol Cell.

[6] Mayer C, Zhao J, Yuan X, Grummt I (2004). mTOR-dependent activation of the transcription factor TIF-IA links rRNA synthesis to nutrient availability. Genes Dev.

[7] Drygin D, Rice WG, Grummt I (2010). The RNA polymerase I transcription machinery: an emerging target for the treatment of cancer. Annu Rev Pharm Toxicol.

[8] Perry RP, Kelley DE (1970). Inhibition of RNA synthesis by actinomycin D: characteristic dose-response of different RNA species. J Cell Physiol.

[9] Xu H, Di Antonio M, McKinney S, Mathew V, Ho B, O’Neil NJ (2017). CX-5461 is a DNA G-quadruplex stabilizer with selective lethality in BRCA1/2 deficient tumours. Nat Commun.

[10] Quin J, Chan KT, Devlin JR, Cameron DP, Diesch J, Cullinane C (2016). Inhibition of RNA polymerase I transcription initiation by CX-5461 activates non-canonical ATM/ATR signaling. Oncotarget.

[11] Colis L, Peltonen K, Sirajuddin P, Liu H, Sanders S, Ernst G (2014). DNA intercalator BMH-21 inhibits RNA polymerase I independent of DNA damage response. Oncotarget.

[12] Peltonen K, Colis L, Liu H, Trivedi R, Moubarek MS, Moore HM (2014). A targeting modality for destruction of RNA polymerase I that possesses anticancer activity. Cancer Cell.

[13] Bruno PM, Liu Y, Park GY, Murai J, Koch CE, Eisen TJ (2017). A subset of platinum-containing chemotherapeutic agents kills cells by inducing ribosome biogenesis stress. Nat Med.

[14] Pushpakom S, Iorio F, Eyers PA, Escott KJ, Hopper S, Wells A (2019). Drug repurposing: progress, challenges and recommendations. Nat Rev Drug Discov.

[15] Skrott Z, Mistrik M, Andersen KK, Friis S, Majera D, Gursky J (2017). Alcohol-abuse drug disulfiram targets cancer via p97 segregase adaptor NPL4. Nature.

[16] Peltonen K, Colis L, Liu H, Jaamaa S, Moore HM, Enback J (2010). Identification of novel p53 pathway activating small-molecule compounds reveals unexpected similarities with known therapeutic agents. PLoS One.

[17] Pasquier B (2016). Autophagy inhibitors. Cell Mol Life Sci.

[18] Qiao S, Tao S, Rojo de la Vega M, Park SL, Vonderfecht AA, Jacobs SL (2013). The antimalarial amodiaquine causes autophagic-lysosomal and proliferative blockade sensitizing human melanoma cells to starvation- and chemotherapy-induced cell death. Autophagy.

[19] Sohn TA, Bansal R, Su GH, Murphy KM, Kern SE (2002). High-throughput measurement of the Tp53 response to anticancer drugs and random compounds using a stably integrated Tp53-responsive luciferase reporter. Carcinogenesis.

[20] Carpenter AE, Jones TR, Lamprecht MR, Clarke C, Kang IH, Friman O (2006). CellProfiler: image analysis software for identifying and quantifying cell phenotypes. Genome Biol.

[21] Tse WC, Boger DL (2004). A fluorescent intercalator displacement assay for establishing DNA binding selectivity and affinity. Acc Chem Res.

[22] Kwon I, Xiang S, Kato M, Wu L, Theodoropoulos P, Wang T (2014). Poly-dipeptides encoded by the C9orf72 repeats bind nucleoli, impede RNA biogenesis, and kill cells. Science.

[23] Bindea G, Mlecnik B, Hackl H, Charoentong P, Tosolini M, Kirilovsky A (2009). ClueGO: a Cytoscape plug-in to decipher functionally grouped gene ontology and pathway annotation networks. Bioinformatics.

[24] Herdman C, Mars JC, Stefanovsky VY, Tremblay MG, Sabourin-Felix M, Lindsay H (2017). A unique enhancer boundary complex on the mouse ribosomal RNA genes persists after loss of Rrn3 or UBF and the inactivation of RNA polymerase I transcription. PLoS Genet.

[25] Peltonen K, Colis L, Liu H, Jaamaa S, Zhang Z, Af Hallstrom T (2014). Small molecule BMH-compounds that inhibit RNA polymerase I and cause nucleolar stress. Mol Cancer Ther.

[26] Boulon S, Westman BJ, Hutten S, Boisvert FM, Lamond AI (2010). The nucleolus under stress. Mol Cell.

[27] Lindstrom MS, Jurada D, Bursac S, Orsolic I, Bartek J, Volarevic S (2018). Nucleolus as an emerging hub in maintenance of genome stability and cancer pathogenesis. Oncogene.

[28] Floutsakou I, Agrawal S, Nguyen TT, Seoighe C, Ganley AR, McStay B (2013). The shared genomic architecture of human nucleolar organizer regions. Genome Res.

[29] Daniely Y, Borowiec JA (2000). Formation of a complex between nucleolin and replication protein A after cell stress prevents initiation of DNA replication. J Cell Biol.

[30] Calo E, Flynn RA, Martin L, Spitale RC, Chang HY, Wysocka J (2015). RNA helicase DDX21 coordinates transcription and ribosomal RNA processing. Nature.

[31] Trere D (2000). AgNOR staining and quantification. Micron.

[32] Horn HF, Vousden KH (2008). Cooperation between the ribosomal proteins L5 and L11 in the p53 pathway. Oncogene.

[33] Kruhlak M, Crouch EE, Orlov M, Montano C, Gorski SA, Nussenzweig A (2007). The ATM repair pathway inhibits RNA polymerase I transcription in response to chromosome breaks. Nature.

[34] Larsen DH, Hari F, Clapperton JA, Gwerder M, Gutsche K, Altmeyer M (2014). The NBS1-Treacle complex controls ribosomal RNA transcription in response to DNA damage. Nat Cell Biol.

[35] Lyu YL, Kerrigan JE, Lin CP, Azarova AM, Tsai YC, Ban Y (2007). Topoisomerase IIbeta mediated DNA double-strand breaks: implications in doxorubicin cardiotoxicity and prevention by dexrazoxane. Cancer Res.

[36] Burger K, Muhl B, Harasim T, Rohrmoser M, Malamoussi A, Orban M (2010). Chemotherapeutic drugs inhibit ribosome biogenesis at various levels. J Biol Chem.

[37] Harding SM, Boiarsky JA, Greenberg RA (2015). ATM dependent silencing links nucleolar chromatin reorganization to DNA damage recognition. Cell Rep.

[38] Mauthe M, Orhon I, Rocchi C, Zhou X, Luhr M, Hijlkema KJ (2018). Chloroquine inhibits autophagic flux by decreasing autophagosome-lysosome fusion. Autophagy.

[39] McAfee Q, Zhang Z, Samanta A, Levi SM, Ma XH, Piao S (2012). Autophagy inhibitor Lys05 has single-agent antitumor activity and reproduces the phenotype of a genetic autophagy deficiency. Proc Natl Acad Sci USA.

[40] Lamb J, Crawford ED, Peck D, Modell JW, Blat IC, Wrobel MJ (2006). The Connectivity Map: using gene-expression signatures to connect small molecules, genes, and disease. Science.

[41] King MA, Ganley IG, Flemington V (2016). Inhibition of cholesterol metabolism underlies synergy between mTOR pathway inhibition and chloroquine in bladder cancer cells. Oncogene.

[42] Maes H, Kuchnio A, Peric A, Moens S, Nys K, De Bock K (2014). Tumor vessel normalization by chloroquine independent of autophagy. Cancer Cell.

[43] Hetz C, Papa FR (2018). The unfolded protein response and cell fate control. Mol Cell.

[44] Gallagher LE, Radhi OA, Abdullah MO, McCluskey AG, Boyd M, Chan EYW (2017). Lysosomotropism depends on glucose: a chloroquine resistance mechanism. Cell Death Dis.

[45] WHO. Guidelines for the treatment of malaria—3rd ed. Global Malaria Programme. Geneva: WHO; 2015.

[46] White NJ (1996). Can amodiaquine be resurrected?. Lancet.

[47] Shimizu S, Atsumi R, Itokawa K, Iwasaki M, Aoki T, Ono C (2009). Metabolism-dependent hepatotoxicity of amodiaquine in glutathione-depleted mice. Arch Toxicol.

[48] Lobach AR, Uetrecht J (2014). Involvement of myeloperoxidase and NADPH oxidase in the covalent binding of amodiaquine and clozapine to neutrophils: implications for drug-induced agranulocytosis. Chem Res Toxicol.

[49] Maggs JL, Tingle MD, Kitteringham NR, Park BK (1988). Drug-protein conjugates–XIV. Mechanisms of formation of protein-arylating intermediates from amodiaquine, a myelotoxin and hepatotoxin in man. Biochem Pharmacol.

[50] Li XQ, Bjorkman A, Andersson TB, Ridderstrom M, Masimirembwa CM (2002). Amodiaquine clearance and its metabolism to N-desethylamodiaquine is mediated by CYP2C8: a new high affinity and turnover enzyme-specific probe substrate. J Pharm Exp Ther.

[51] Zhang Y, Vermeulen NP, Commandeur JN (2017). Characterization of human cytochrome P450 mediated bioactivation of amodiaquine and its major metabolite N-desethylamodiaquine. Br J Clin Pharmcol.

[52] Westerink WM, Schoonen WG (2007). Cytochrome P450 enzyme levels in HepG2 cells and cryopreserved primary human hepatocytes and their induction in HepG2 cells. Toxicol Vitr.

[53] Zhang Y, den Braver-Sewradj SP, den Braver MW, Hiemstra S, Vermeulen NPE, van de Water B (2018). Glutathione S-transferase P1 protects against amodiaquine quinoneimines-induced cytotoxicity but does not prevent activation of endoplasmic reticulum stress in HepG2 cells. Front Pharm.

[54] Walsky RL, Gaman EA, Obach RS (2005). Examination of 209 drugs for inhibition of cytochrome P450 2C8. J Clin Pharmacol.

[55] Zhang W, Ramamoorthy Y, Kilicarslan T, Nolte H, Tyndale RF, Sellers EM (2002). Inhibition of cytochromes P450 by antifungal imidazole derivatives. Drug Metab Dispos.

[56] Morgado-Palacin L, Llanos S, Urbano-Cuadrado M, Blanco-Aparicio C, Megias D, Pastor J (2014). Non-genotoxic activation of p53 through the RPL11-dependent ribosomal stress pathway. Carcinogenesis.

[57] Hein N, Cameron DP, Hannan KM, Nguyen NN, Fong CY, Sornkom J (2017). Inhibition of Pol I transcription treats murine and human AML by targeting the leukemia-initiating cell population. Blood.

[58] Cornelison R, Dobbin ZC, Katre AA, Jeong DH, Zhang Y, Chen D (2017). Targeting RNA-polymerase I in both chemosensitive and chemoresistant populations in epithelial ovarian cancer. Clin Cancer Res.

[59] Lawrence MG, Obinata D, Sandhu S, Selth LA, Wong SQ, Porter LH (2018). Patient-derived models of abiraterone- and enzalutamide-resistant prostate cancer reveal sensitivity to ribosome-directed therapy. Eur Urol.

[60] Poortinga G, Quinn LM, Hannan RD (2015). Targeting RNA polymerase I to treat MYC-driven cancer. Oncogene.

[61] Verbaanderd C, Maes H, Schaaf MB, Sukhatme VP, Pantziarka P, Sukhatme V (2017). Repurposing drugs in oncology (ReDO)-chloroquine and hydroxychloroquine as anti-cancer agents. Ecancermedicalscience.

