# Supplementary material for: The antimalarial drug amodiaquine stabilizes p53 through ribosome biogenesis stress, independently of its autophagy-inhibitory activity
Source: Cell Death Differ. 2019 Jul 8;27(2):773–89. doi: 10.1038/s41418-019-0387-5 (PMC7205879; doi:10.1038/s41418-019-0387-5)
Supplement: Supplementary file 1 — Supplementary material [file 41418_2019_387_MOESM1_ESM.pdf]

Supplementary information

The antimalarial drug amodiaquine stabilizes p53 through ribosome biogenesis stress, independently of its autophagy-inhibitory activity

Espinoza *et al,* Cell Death & Differentiation, 2019

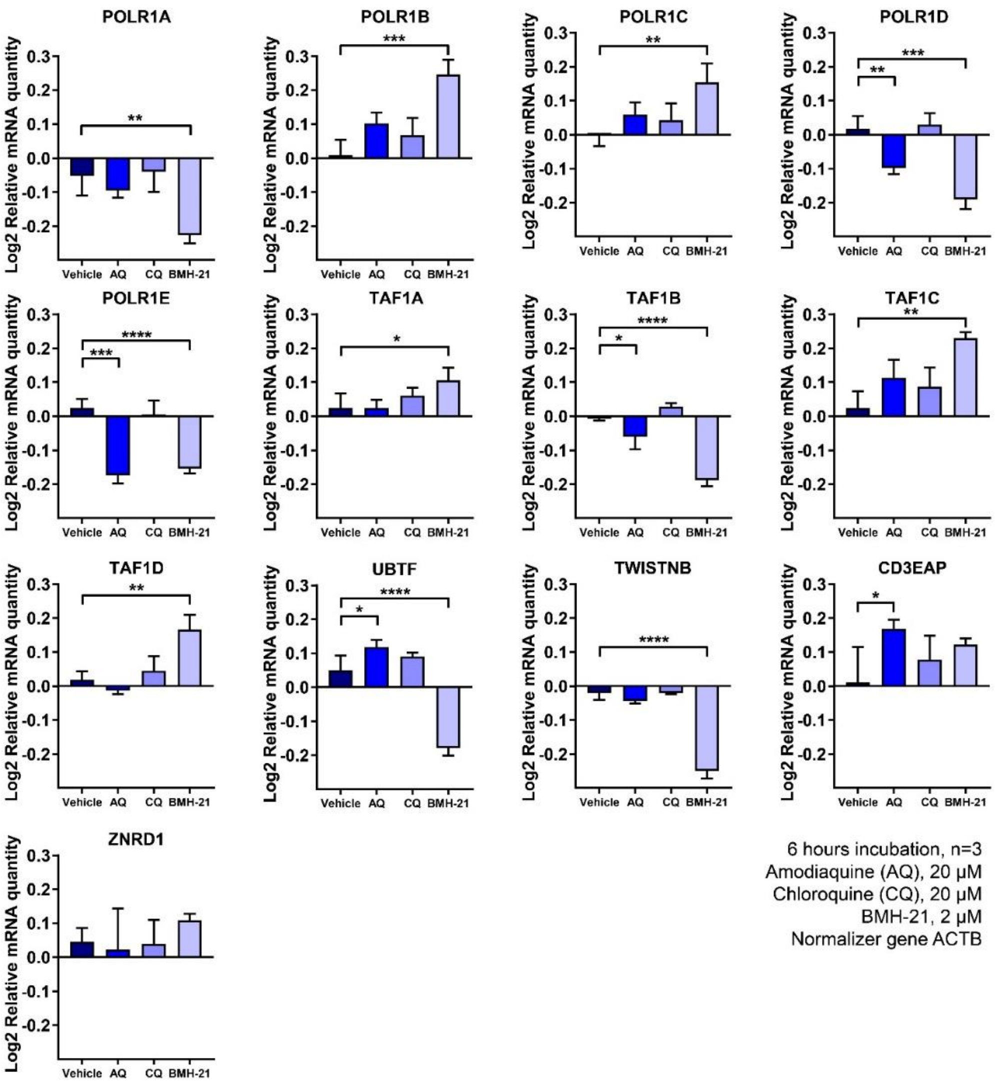

Supplementary Figure 1. Transcriptional changes of genes that structure the preinitiation complex and RNA Pol I complex. mRNA levels were assessed in U2OS cells after exposure to CQ, AQ and BMH-21 during 6 hours. Data shown as mean ± SD of triplicate wells and are representative of triplicate treatments; Statistical significance was calculated by one-way ANOVA using log- transformed data and Dunnett's multiple test comparison (* $p<0.05$, ** $p<0.01$, *** $p<0.001$, **** $p<0.001$)

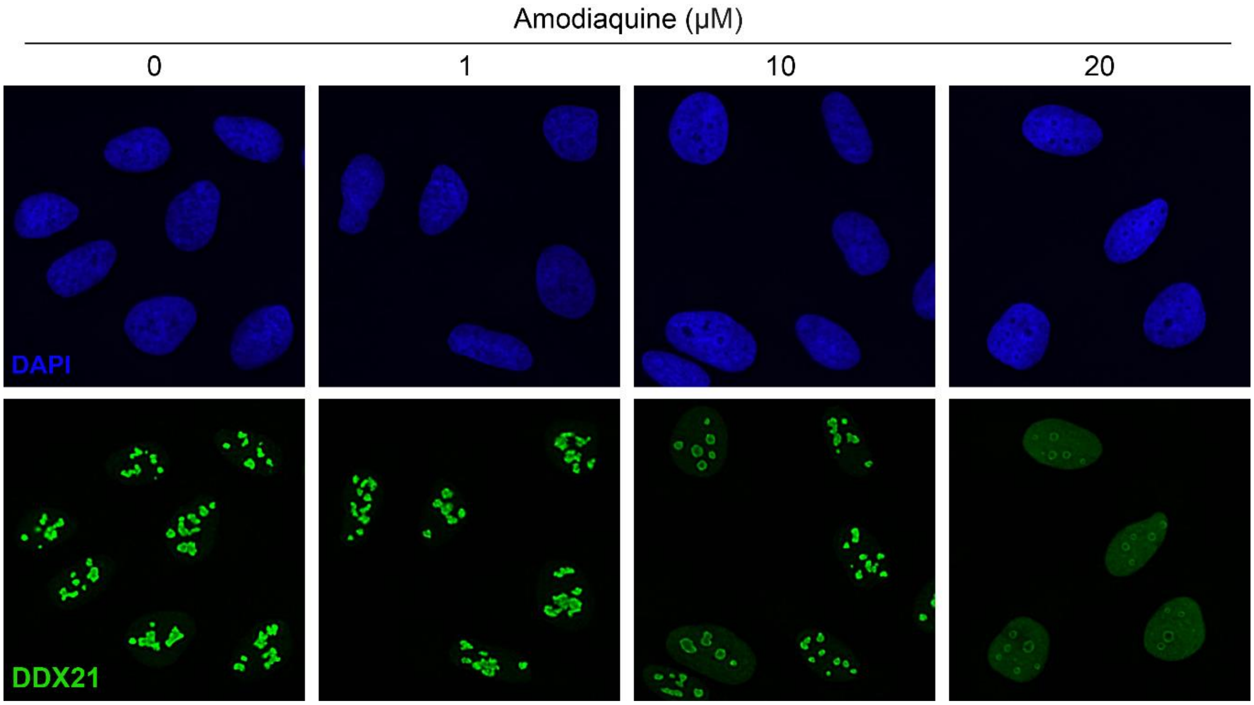

Supplementary Figure 2. **Amodiaquine treatment causes RNA helicase DDX21 translocation into the nucleoplasm**. Immunofluorescence analysis of U2OS cells treated with increasing doses of AQ (6 hours) and immunostained for DDX21.

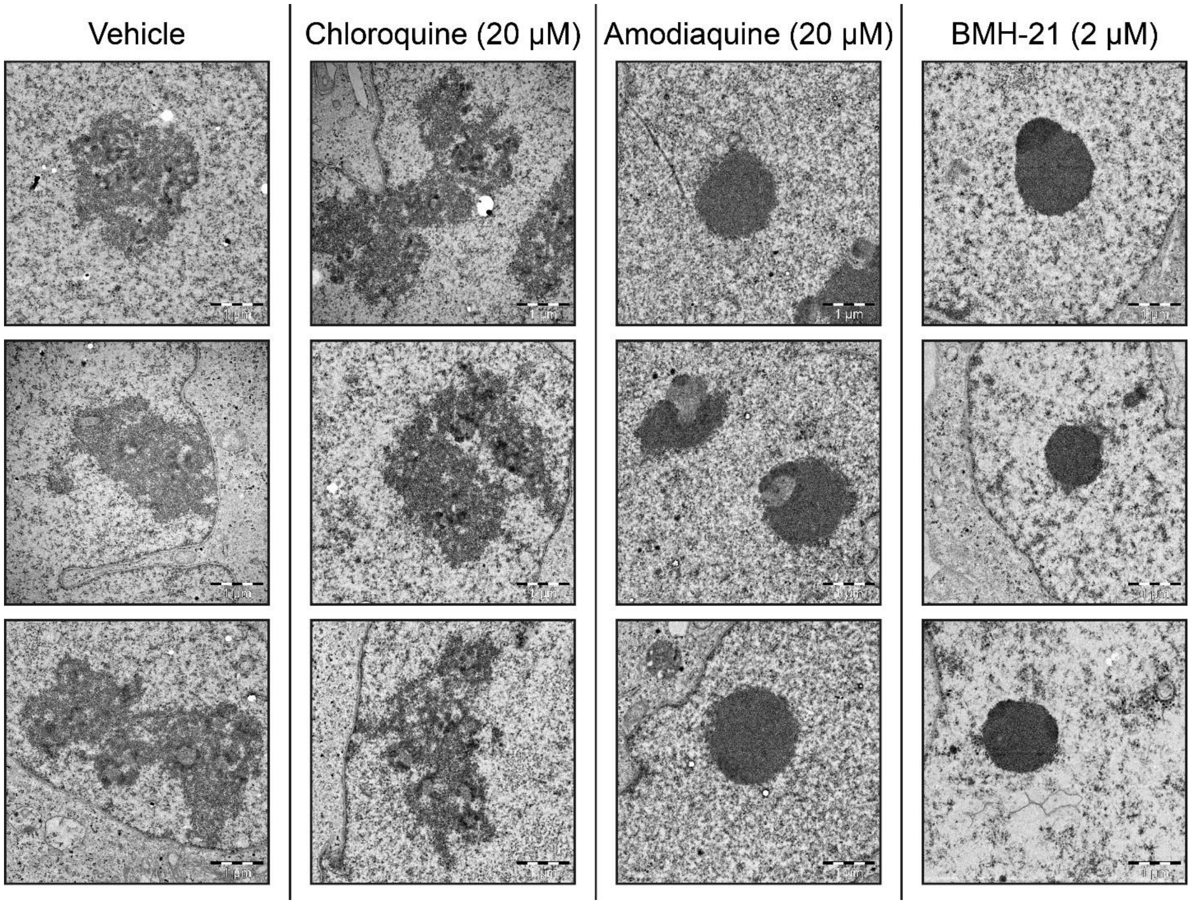

Supplementary Figure 3. Additional images showing the ultrastructural changes in the nucleolus after exposure to CQ, AQ and BHM-21 during 6 hours (Bar= 1 μM).

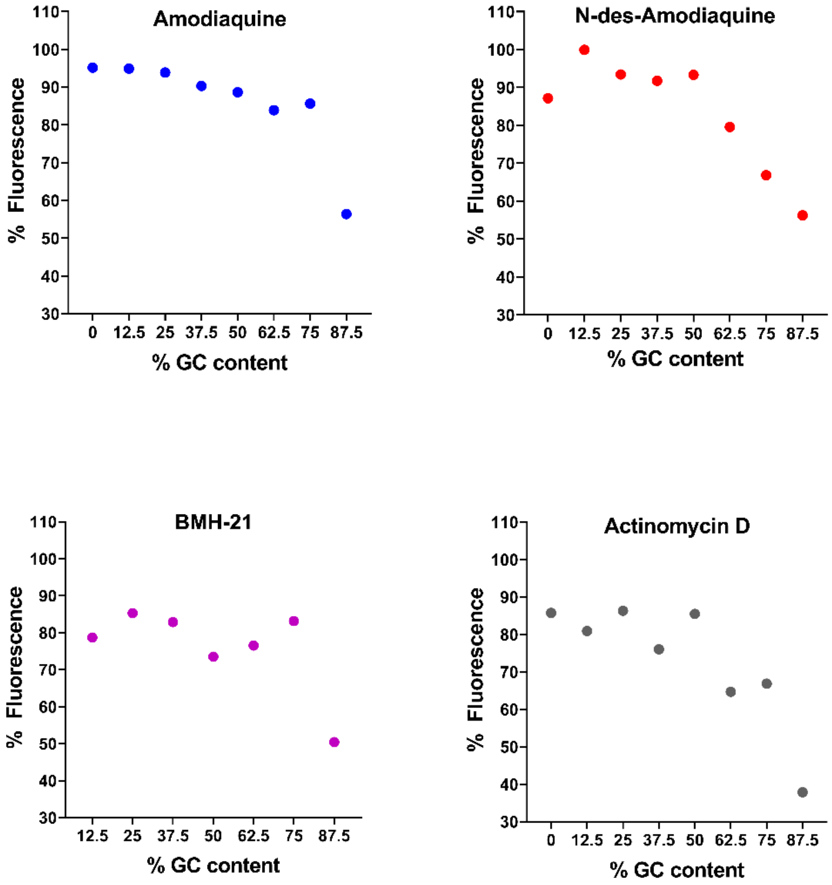

Supplementary Figure 4. Fluorescent intercalator displacement (FID) assay of amodiaquine, N-desethyl-amodiaquine, BMH-21 and actinomycin D. Plots show percentage of fluorescence according to the GC content.

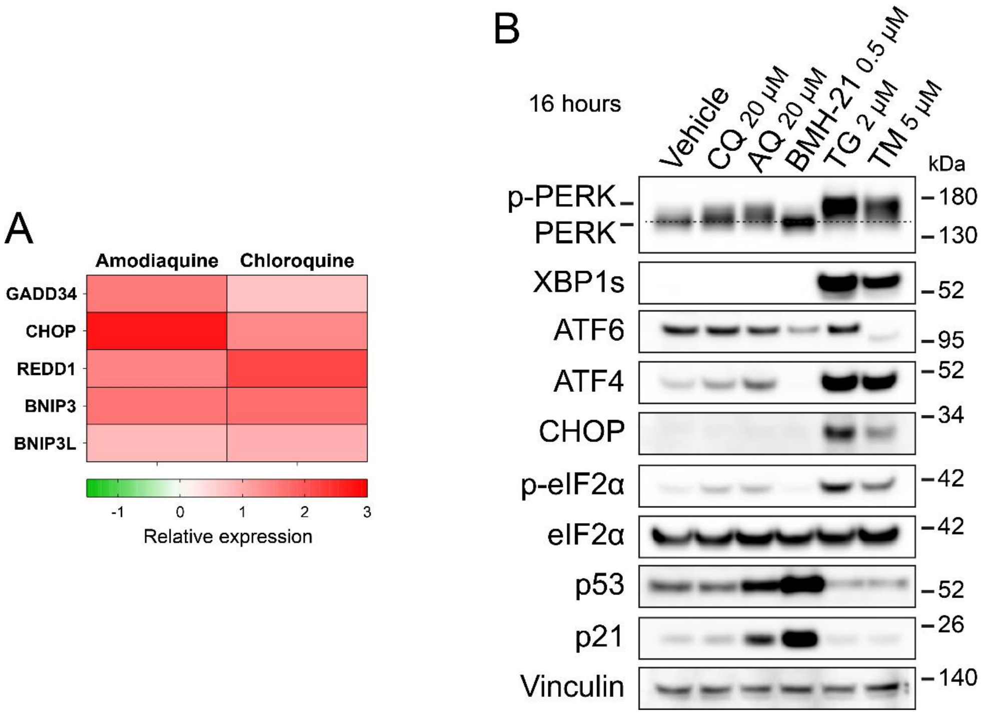

Supplementary Figure 5. Amodiaquine and chloroquine induce a mild ER-stress. A) Amodiaquine and chloroquine induce expression of ER-stress related genes; RNAseq data, relative expression compared to untreated control. B) Immunoblot analysis of ER stress markers. U2OS cells were treated during 16 hours with the compounds using the indicated concentrations. PERK hyperphosphorylation is observed as a shift in molecular size. Thapsigargin (TG) and Tunicamycin (TM) were used as positive controls of ER stress. CQ=chloroquine; AQ=Amodiaquine.

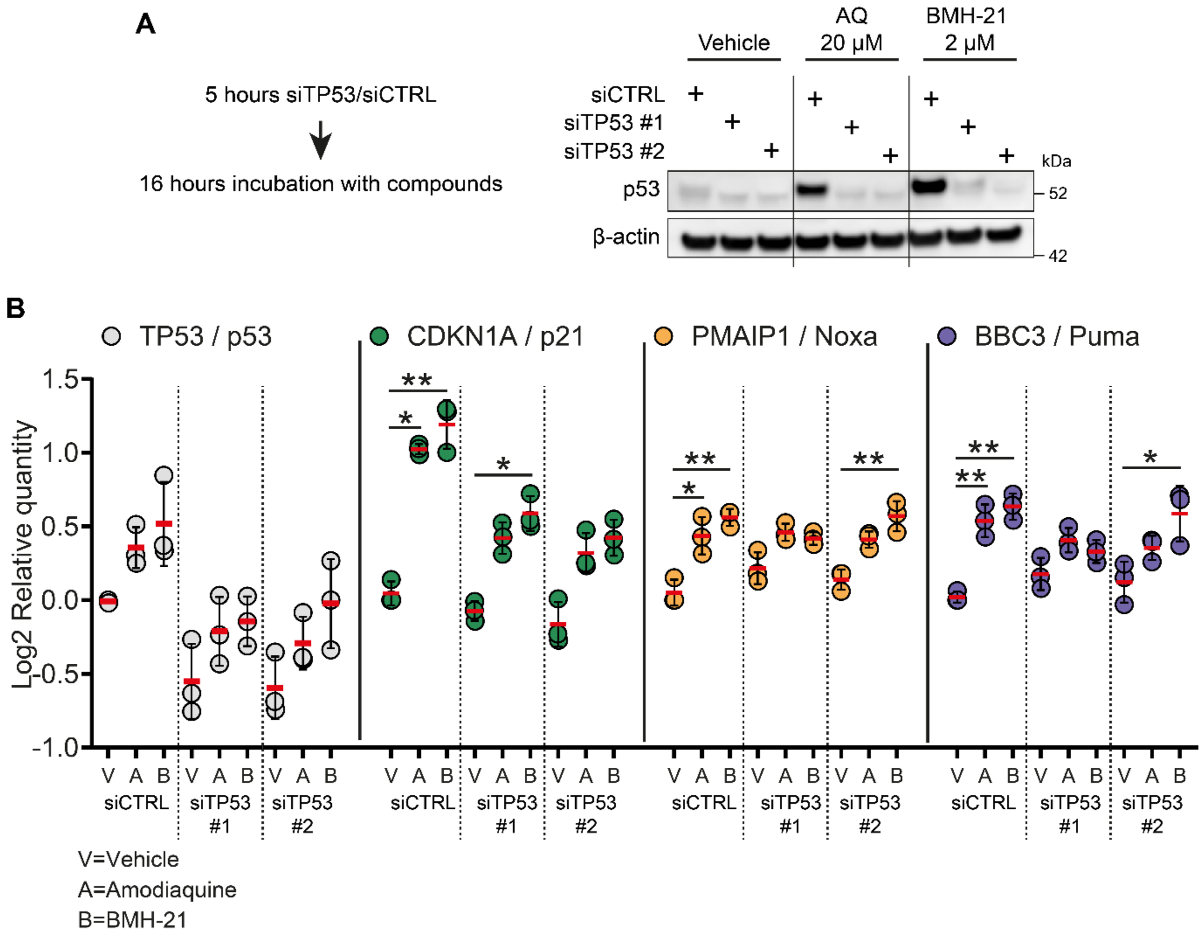

Supplementary Figure 6. Expression of p53-regulated genes induced by Amodiaquine and BMH-21. A) U2OS cells were treated with 20 nM siRNA during 5 hours prior to treatment with Amodiaquine and BMH-21. Immunoblot analysis showed no p53 activation after 16 hours of incubation with compounds in siTP53 treated cells. B) RT-qPCR analysis of TP53, CDKN1A, PMAIP1 and BBC3 mRNAs in U2OS cells treated as in A.

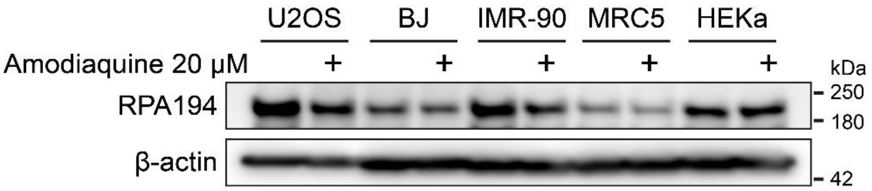

Supplementary Figure 7. RPA194 degradation in non-transformed cells treated with amodiaquine. Immunoblot analysis of RPA194 in BJ fibroblasts, IMR-90 fibroblasts, MRC5 fibroblasts and HEKa Primary Epidermal Keratinocytes treated with amodiaquine for 6 hours. U2OS cancer cell line is included as control.

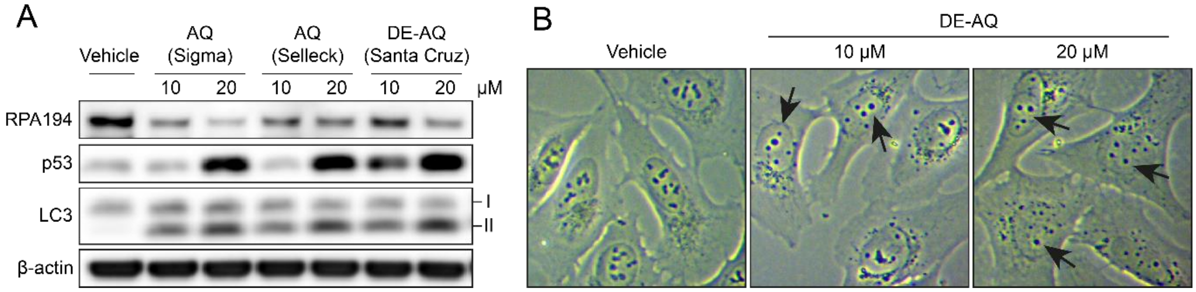

Supplementary Figure 8. The DE-AQ metabolite has similar nucleolar disruptive activity as AQ. A) DE-AQ induces RPA194 degradation, activation of p53 and accumulation of LC3-II. U2OS cells were treated during 6 hours with the compounds. B) Brightfield images of U2OS cells treated with DE-AQ. Black arrows show nucleoli with altered morphology.

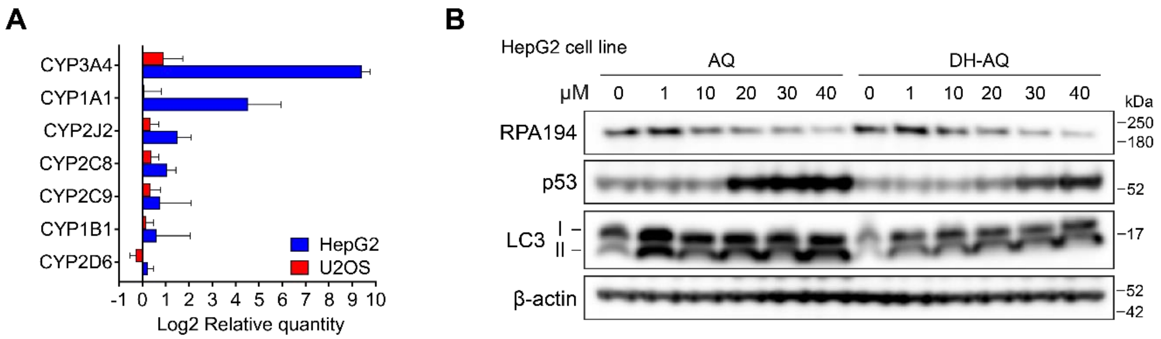

Supplementary Figure 9. A) HepG2 expresses higher levels of CYP genes than U2OS assessed by RT-qPCR analysis, (n=3). B) Immunoblot analysis of RPA194, p53 and LC3 in HepG2 cells treated with increasing doses of AQ and its non-reactive analog DH-AQ during 6 hours.

Supplementary table S1. Cell lines

Cell line	Tissue of origin	Species	Medium
U2OS	Sarcoma	Human	DMEM 10% FBS
MEF	Fibroblast	Mouse	DMEM 10% FBS
BJ	Fibroblast	Human	DMEM 10% FBS
MRC5	Fibroblast	Human	DMEM 10% FBS
IMR-90	Fibroblast	Human	DMEM 10% FBS
HepG2	Hepatic cancer	Human	DMEM 10% FBS
A375	Melanoma	Human	DMEM 10% FBS
A549	Lung carcinoma	Human	DMEM 10% FBS
MCF7	Breast adenocarcinoma	Human	DMEM 10% FBS
HT-29	Colorectal adenocarcinoma	Human	DMEM 10% FBS
RKO	Colorectal adenocarcinoma	Human	DMEM 10% FBS
RKO ATM -/- 2.1	Colorectal adenocarcinoma	Human	DMEM 10% FBS
RKO ATM -/- 6.3	Colorectal adenocarcinoma	Human	DMEM 10% FBS
SK-CO-1	Colorectal adenocarcinoma	Human	DMEM 10% FBS
SW948	Colorectal adenocarcinoma	Human	DMEM 10% FBS
SW837	Colorectal adenocarcinoma	Human	DMEM 10% FBS
SW1116	Colorectal adenocarcinoma	Human	DMEM 10% FBS
T84	Colorectal adenocarcinoma	Human	DMEM 10% FBS
LoVo	Colorectal adenocarcinoma	Human	DMEM 10% FBS
HCT116	Colorectal adenocarcinoma	Human	DMEM 10% FBS
LS123	Colorectal adenocarcinoma	Human	DMEM 10% FBS
HEKa	Primary Epidermal Keratinocytes	Human	Dermal Cell Basal Medium + Keratinocyte Growth Kit (ATCC, PCS-200-040

Supplementary table S2. Antibodies

Antibodies	Company	Clone	Catalogue No	Application (dilution)
RPA194 (POLR1A) C-1	Santa Cruz	C-1	sc-48385	WB (1:2,000); IF (1:500); Wes (1:50)
p53,	Abcam	DO-1	ab1101	WB (1:2,000): Wes (1:50)
p21	Cell Signaling	DCS60	2946	WB (1:1,000)
Beclin-1	Cell Signaling	D4OC5	3495	WB (1:2,000)
p62 (SQSTM1)	Cell Signaling	D5L7G	88588	WB (1:2,000)
p62 (SQSTM1)	Santa Cruz	D-3	sc-28359	WB (1:2,000)
LC3B (D11) XP®	Cell Signaling	D11	3868	IF (1:500)
LC3B	Cell Signaling	Polyclonal	2775S	WB (1:2,000)
β-actin	Abcam	AC-15	ab6276	WB (1:20,000); Wes (1:150)
phospho-H2AX (ser139)	Millipore	JBW301	05-636	WB (1:1,000) / IF (1:500)
ATM	Abcam	2C1	ab78	WB (1:1,000)
phospho-ATM (Ser1981)	Abcam	D6H9	5883S	WB (1:1,000)
Nucleolin	Abcam	Polyclonal	ab22758	WB (1:2,000) / IF (1:500)
RPL5	Bethyl Laboratories	Polyclonal	A303-933A	WB (1:2,000)
RPL11	Thermo	3A4A7	37-3000	WB (1:500)
LAMP1	Cell Signaling	D4O1S	15665	IF (1:500)
LAMP1	Cell Signaling	C54H11	3243S	WB (1:1,000)
GAPDH	Millipore	Polyclonal	AB2302	WB (1:5,000)
DDX21	Novus Biologicals	Polyclonal	NBP1-83310	IF (1:500)
KAP1	Abcam	20C1	ab22553	WB (1:1,000)
phospho-KAP1 (Ser824)	Abcam	EPR5248	ab133440	WB (1:1,000)
UBF	Santa Cruz	F-9	sc-13125	IF (1:500)
Fibrillarin	Abcam	Polyclonal	ab5821	IF (1:500)
Anti-amodiaquine	Thermo	6D10	HYB 320-04-02	Wes (1:25)
PERK	Cell Signaling	D11A8	5683	WB (1:1,000)
XBP-1s	Cell Signaling	D2C1F	12782	WB (1:1,000)
ATF6	Cell Signaling	D4Z8V	65880	WB (1:1,000)
ATF4	Cell Signaling	D4B8	11815	WB (1:1,000)
CHOP	Cell Signaling	D46F1	5554	WB (1:1,000)
Phosphor-eIF2α (Ser51)	Cell Signaling	Polyclonal	9721	WB (1:1,000)
eIF2α	Cell Signaling	Polyclonal	9722	WB (1:1,000)
Vinculin	Abcam	EPR8185	ab129002	WB (1:1,000)
Anti-chicken HRP	Thermo	Polyclonal	A16054	WB / 1:5000
Anti-mouse Alexa-647	Thermo	Polyclonal	A-21235	IF / 1:1000
Anti-mouse Alexa-488	Thermo	Polyclonal	A-11029	IF / 1:1000
Anti-rabbit Alexa-647	Thermo	Polyclonal	A-21244	IF / 1:1000
Anti-rabbit Alexa-488	Thermo	Polyclonal	A-11008	IF / 1:1000
Goat Anti-Mouse IgG (Cy3)	Abcam	Polyclonal	ab97035	WB / 1:1000
Goat Anti-Rabbit IgG (Cy5)	Abcam	Polyclonal	ab97077	WB / 1:1000

Supplementary table S3. Chemicals

Chemical	Company	Catalogue code	Solvent
Amodiaquin dihydrochloride dihydrate	Sigma-Aldrich	A2799	Water
Amodiaquin dihydrochloride dihydrate	Selleck Chemicals	S4589	Water
N-Desethyl Amodiaquine Hydrochloride	Santa Cruz Biotechnology	sc-212178	Water
Chloroquine diphosphate salt	Sigma-Aldrich	C6628	Water
Hydroxychloroquine sulfate	Sigma-Aldrich	H0915	Water
Doxorubicin	Sigma-Aldrich	D1515	DMSO
KU-60019	Selleck Chemicals	S1570	DMSO
Aloxistatin	Selleck Chemicals	S7393	DMSO
Pepstatin A	Selleck Chemicals	S7381	DMSO
Bafilomycin A1	Selleck Chemicals	S1413	DMSO
Lys05 trihydrochloride	MedKoo Biosciences	406969	DMSO
BMH-21	Sigma-Aldrich	SML1183	DMSO
MG-132	Selleck Chemicals	S2619	DMSO
CX-5461	Cellagen Technologies	C2954	DMSO
Cycloheximide	Sigma-Aldrich	C7698	DMSO
Actinomycin D	Sigma-Aldrich	A1410	DMSO
Thapsigargin	Abcam	ab120286	DMSO
Tunicamycin	Cayman chemicals	11445	DMSO
Clotrimazole	Sigma-Aldrich	C6019	DMSO

Supplementary table S4. RT-qPCR primer sequences

Target	Forward	Reverse
47S_5´ETS*	GAACGGTGGTGTGTCGTT	GCGTCTCGTCTCGTCTCACT
18S_5’-junction*	GCCGCGCTCTACCTTACCTACCT	CAGACATGCATGGCTTAATCTTTG
18S_3’-junction*	AGTCGTAACAAGGTTTCCGTAGGT	CCTCCGGGCTCCGTTAAT
5.8S_5’-junction*	TACGACTCTTAGCGGTGGATCA	TCACATTAATTCTCGCAGCTAGCT
5.8S_3’-junction*	GAATTGCAGGACACATTGATCATC	GGCAAGCGACGCTCAGA
28S_5’-junction*	CCGAGACGCGACCTCAGAT	TCCGCTGACTAATATGCTTAAATTCA
18S*	GATGGTAGTCGCCGTGCC	GCCTGCTGCCTTCCTTGG
5.8S*	ACTCGGCTCGTGCGTC	GCGACGCTCAGACAGG
28S*	GTGACGCGCATGAATGGA	TGTGGTTTCGCTGGATAGTAGGT
GAPDH	ACCTGACCTGCCGTCTAGAAAA	CAAAGTGGTCGTTGAGGGCAAT
ACTB	TCACAATGTGGCCGAGGACTTT	AGAAGTGGGGTGGCTTTTAGGA
POLR1A	TTTGCCGTGTATGGCATCGC	TGTCATCTGCTGTAGCGGGG
POLR1B	TGAGTGGGGCGGAATATGGG	CTGCGGTTTCGGTTTCTCGG
POLR1C	TTGCCCGGGTTCGAGATCATT	TGATGGCTTCACTCACCAGCA
POLR1D	TGCACGAGGAAGACCATACCC	ACAGCTGGAAGGGTACCTCG
POLR1E	GTGACTGCTCTGGTCAGCGA	ACACGTCTTCAGGCTTGGCT
TAF1A	GCAGGAGGAGTTACGAGCCG	TCCGGCCCACGTGAAGAAAT
TAF1B	CCCCGTCAGCAAAGCATCAC	GGCAAGTGTCTGTGGCATGG
TAF1C	CTCCCTCACTGGCTGACCTG	CTGCATTGCCTGCAGAAGGG
TAF1D	TGGGCCACGGAAATAGCCAA	CTGGCCTGGTGTCCTAGAGC
UBTF	TCATAAGGCCCTGGAGCAGC	ACTTGTCCTTGAGCTGGCGT
TWISTNB	GAGTTGCCGACTTATGCCGC	CTGTTCTCGAATGCCGGTGC
CD3EAP	GCTCCCGGATGATGCCCTAC	AACCGAGCAGCATCCTCACC
ZNRD1	AACTCCCTCCTCAGACCCGA	GTGACCGTATCCTGAGCCCC
CYP3A4	AGCACCGAGTGGATTTCCTTCAG	GCTCCAGATCGGACAGAGCTTT
CYP1A1	TACCTACCCAACCCTTCCCTGAAT	TCAATCAGGCTGTCTGTGATGTCC
CYP2J2	CATCACCTTCGGAGAACGCT	GGGTCCAGGCAGGAATTTCA
CYP2C8	CTTGGTTGGCACTGTAGCTGATCT	TCCTGGACTTTAGCTGTGACCTCT
CYP2C9	TCTATGGCCCTGTGTTCACTCTG	CAGGGCTTCCTTCACTGCTTCATA
CYP1B1	ACCAGGTATCCTGATGTGCAGACT	GCTTCATAAAGGAAGGCCAGGACA
CYP2D6	ATTCATGAGGTGCAGCGCTTTG	CTCATCCTTCAGCACCGATGACA
TP53 (p53)	CAGTTGGGCAGCTGGTTAGG	ATCCTCCAGGGTGTGGGATG
CDKN2A (p21)	AATCGTCCAGCGACCTTCCT	CTGACTCCTTGTTCCGCTGC
PMAIP1 (Noxa)	TACCGCTGGCCTACTGTGAA	ATGTGCTGAGTTGGCACTGA
BBC3 (Puma)	GCGATTGCGATTGGGTGAGA	TACTTCCTGCCCTGCTCTGG

*Sequences obtained from Kwon *et al*, 2014.

Supplementary table S5. ChIP-qPCR primer sequences

rDNA region	Position	Forward	Position	Reverse
upstream	-988	GCTTCTCGACTCACGGTTTC	-798	GGAGCTCTGCCTAGCTCACA
upstream	-410	GATCCTTTCTGGCGAGTCC	-272	GGAGCCGGAAGCATTTTC
promoter	-48	GAGGTATATCTTTCGCTCCGAGTC	-14	CAGCAATAACCCGGCGG
promoter	-46	GGTATATCTTTCGCTCCGAG	13	AGCGACAGGTCGCCAGAGGA
5'ETS	851	GAACGGTGGTGTGTCGTT	961	GCGTCTCGTCTCGTCTCACT
5'ETS	1297	CAGGTGTTTCCTCGTACCG	1483	GCTACCATAACGGAGGCAGA
18S	4013	AAACGGCTACCACATCCAAG	4148	CCTCCAATGGATCCTCGTTA
18S	4446	CCCGAAGCGTTTACTTTGAA	4612	CGGTCCAAGAATTTCACCTA
28S	10319	GAACTTTGAAGGCCGAAGTG	10450	ATCTGAACCCGACTCCCTTT
28S	12293	TGGGTTTTAAGCAGGAGGTG	12472	AACCTGTCTCACGACGGTCT
IGS	18449	TGGTGGGATTGGTCTCTCTC	18572	CAGCCTGCGTACTGTGAAAA
IGS	30541	ACTGGCGAGTTGATTTCTGG	30621	CGAGACAGTCGAGGGAGAAG

*Sequences obtained from Peltonen *et al*, 2014.

Supplementary table S6. FID oligo hairpins

Sequence (5'-3')	GC content (%)
TTTATATAAAAAATATATAAA	0
CAATAAATAAAAAATTTATTG	12.5
CATAATTCAAAAAGAATTATG	25
CGAATAACAAAAAGTTATTCG	37.5
CGAGAATCAAAAACATTCTCG	50
CGATGCACAAAAAGTGCATCG	62.5
CGCGAACCAAAAAGGTTCGCG	75
CGGCCTGCAAAAAGCAGGCCG	87.5
